# Childhood Cancer: Occurrence, Treatment and Risk of Second Primary Malignancies

**DOI:** 10.3390/cancers13112607

**Published:** 2021-05-26

**Authors:** Sebastian Zahnreich, Heinz Schmidberger

**Affiliations:** Department of Radiation Oncology and Radiation Therapy, University Medical Centre of the Johannes Gutenberg University, 55131 Mainz, Germany; heinz.schmidberger@unimedizin-mainz.de

**Keywords:** childhood cancer, second primary malignancy, radiotherapy, chemotherapy, targeted therapy, immunotherapy, epidemiology, etiology, late-effects

## Abstract

**Simple Summary:**

Childhood cancers are mostly of unknown etiology and represent devastating diagnoses. The clinical benefits of steadily increasing tumor control and survival rates are countered by severe and fatal health consequences from genotoxic therapies in long-term survivors of pediatric cancers. Among them, iatrogenic second primary malignancies represent the heaviest burden for the patient. Therefore, particularly in pediatric tumor patients, the reduction of genotoxic treatments and the use of targeted or immune-based oncologic strategies are of high clinical interest. The knowledge of therapy-associated as well as intrinsic risk factors for late sequelae of antineoplastic treatments including secondary primary malignancies offers the opportunity to adapt oncologic therapies for high-risk patients and to intensify follow-up with intervention strategies and multidisciplinary care.

**Abstract:**

Cancer represents the leading cause of disease-related death and treatment-associated morbidity in children with an increasing trend in recent decades worldwide. Nevertheless, the 5-year survival of childhood cancer patients has been raised impressively to more than 80% during the past decades, primarily attributed to improved diagnostic technologies and multiagent cytotoxic regimens. This strong benefit of more efficient tumor control and prolonged survival is compromised by an increased risk of adverse and fatal late sequelae. Long-term survivors of pediatric tumors are at the utmost risk for non-carcinogenic late effects such as cardiomyopathies, neurotoxicity, or pneumopathies, as well as the development of secondary primary malignancies as the most detrimental consequence of genotoxic chemo- and radiotherapy. Promising approaches to reducing the risk of adverse late effects in childhood cancer survivors include high precision irradiation techniques like proton radiotherapy or non-genotoxic targeted therapies and immune-based treatments. However, to date, these therapies are rarely used to treat pediatric cancer patients and survival rates, as well as incidences of late effects, have changed little over the past two decades in this population. Here we provide an overview of the epidemiology and etiology of childhood cancers, current developments for their treatment, and therapy-related adverse late health consequences with a special focus on second primary malignancies.

## 1. Introduction

Cancer is the second leading cause of death, with an incidence of about 17 million new cases and 9.6 million cancer-related deaths in 2018 globally [1]. The optimization of conventional antineoplastic therapies and the development of new multimodal oncological strategies as well as diagnostic procedures have significantly improved local and systemic tumor control. Subsequently, the survival rates of cancer patients doubled within the last 40 years, albeit with large variations between different tumor entities. However, this marked clinical benefit for patients is imperiled by iatrogenic long-term health effects primarily induced by the genotoxic impact of the two mainstays of cancer therapy: external beam radiation therapy (EBRT) and chemotherapy (CT).

Besides the eradication of neoplastic cells, genotoxic cancer therapies cause unwanted but inevitable harm to the patients’ healthy tissue associated with deleterious sequelae in long-term cancer survivors including organ dysfunction (e.g., cardiac, pulmonary, or gonadal), impaired growth and development, decreased fertility, compromised cognitive function and second primary malignancies (SPM) as the most deleterious outcome [2,3]. SPMs are an important cause of mortality among cancer survivors and the major determinant of death in patients cured of Hodgkin lymphoma (HL) [4]. In the US, cancer survivors show a 14% higher incidence of new primary tumors compared to the general population representing 18% of total and the 3rd most common tumor diagnosis [5]. The risk for the development of an SPM is subject to lifestyle and genetic factors, the entity and treatment of the primary malignancy, and age at treatment. Survivors of primary cancer in childhood or adolescence are at the ultimate risk for therapy-related late-effects and SPMs due to a higher innate tumor susceptibility determined by genetic factors, genotoxic exposures in a developmental stage, and a long life expectancy. The incidence of primary cancer in children aged 0–14 years is 140.6 per million person-years and in those aged 0–19 years 155.8 per million person-years, representing about 1% of all cancers diagnosed annually worldwide with a gradual increase in recent decades [6,7,8,9]. The average 5-year survival rate of childhood cancer patients improved immensely from less than 30% before 1960 up to 80% to date [10,11]. The effective control of early-onset malignancies by EBRT or multiagent CT elevates the relative risk to develop an SPM up to about 6-fold compared to the general population with an incidence of more than 20% at 30 years after the diagnosis of the primary tumor [12].

Since oncologists face a continuously increasing long-lived population of cancer survivors, there is an urgent need to unravel risk determinants for adverse and fatal late-effects of oncologic therapies, in particular SPMs, and to establish prognostic biomarkers to stratify high-risk patients, adapt their therapies, intensify follow-up and rigorous validation of targets for medical countermeasures. We present a clinical survey of the most prominent childhood cancers, their epidemiology and etiology, past, current, and potentially future advances in treatment as well as risk factors for late effects with the main focus on SPMs.

## 2. Cancer Therapies and Risks of Second Primary Malignancies

The main pillars of cancer therapy are EBRT, CT, and surgery, more recently complemented by targeted therapies against molecular structures of tumor cells and immune-based treatments. To achieve optimal local and systemic tumor control, oncologic strategies were developed from definitive strategies to multimodal options in adjuvant and neoadjuvant settings. Besides the intended eradication of the tumor, cancer therapies are inevitably associated with an exposure of the patient’s healthy tissue that may cause adverse and even fatal side and late effects. 60–90% of childhood cancer survivors develop one or more chronic health conditions and 20–80% experience severe and fatal late sequelae during adulthood [13]. Among them, SPMs represent the most devastating late complications of antineoplastic therapies in cancer survivors. First, we briefly introduce the application and mechanisms of action of anticancer therapies in terms of elimination of neoplastic cell populations, normal tissue toxicities, and SPMs.

### 2.1. External Beam Radiation Therapy

The clinical application of ionizing radiation (IR) in EBRT is used to treat about 60% of all cancer patients [14]. Only minor fractions of patients are treated with brachytherapy or radioisotopes for more specific tumor entities. The genotoxic impact of IR is employed to kill malignant cells or leastwise inactivate their proliferation to terminate clonogenic expansion. However, EBRT is inevitably associated with an exposure of the patient’s normal tissue with primary high in-field doses as well as secondary low out-of-field doses which vary significantly depending on the tumor entity, the applied EBRT technique, radiation quality, and tumor dose.

IR potently induces a large variety of damages in the deoxyribonucleic acid (DNA), such as base damage, DNA cross-links, DNA single-strand breaks, or DNA double-strand breaks, the latter representing the proportionally lowest but most harmful and lethal lesion. The efficiency and fidelity of DNA repair are not only crucial for cellular survival but also determine the fate of the surviving cells and their progeny concerning late sequelae and malignant transformation. Error-prone repair of IR-induced DNA damage can cause transmissible genetic alterations such as genetic and epigenetic mutations or translocations which may foster radiation carcinogenesis [15]. Thus, EBRT is an established risk factor for SPMs which occur at the irradiated sites and preferentially at the tumor margins as sarcomatoid carcinomas [16,17,18]. The IR-associated lifetime risk of SPMs varies widely with age at exposure, sex, and the irradiated tissue or organ with the highest probability for exposure during childhood. Compared to adult cancer patients, the mortality risk from EBRT-related solid SPMs may increase up to 10-fold in pediatric or adolescent patients [19]. Besides SPMs, well-known late consequences of EBRT in pediatric cancer survivors comprise cardiotoxicity, endocrine effects, impact on growth, thyroid and gonadal dysfunction, genitourinary problems, and neurocognitive impairments [20]. Despite a decline in use and dosage during the past decades, EBRT still represents a mainstay for the treatment of pediatric tumors. According to the Surveillance, Epidemiology and End Results 9 (SEER-9) database, a steep reduction in the use of EBRT for pediatric cancers was made for acute lymphoblastic leukemia (ALL), non-Hodgkin lymphoma (NHL), and retinoblastoma (RB) from 57%, 57%, and 30% in 1973–1976 to 11%, 15%, and 2% in 2005–08, respectively. To a lesser extent, EBRT application was also reduced for the brain (70% to 39%), bone (41% to 21%), and Wilms tumors (75% to 53%) as well as neuroblastoma (NB) (60% to 25%). More or less stable application rates were noted for HL (72%), soft tissue cancers (40%), and acute myeloid leukemia (AML, 11%) [21]. Whereas exposure to high cumulative doses during EBRT significantly increases the risk for SPMs in a dose-dependent manner [22,23,24], it is still very much unclear for exposure to low peripheral doses [25]. In general, SPMs are characterized by the following criteria: (i) the SPM arose in the irradiated field after (ii) a latency of more than 4 years, however, in some studies reduced to lower latencies of a few months, (iii) divergent histology than the primary tumor, and (iv) that it developed from normal tissue [26,27].

EBRT aims to deliver high doses of IR to the tumor and spare the healthy tissue as best as possible. Major progress in the protection of the normal tissue during conventional EBRT was achieved by the adaptation and shaping of the primary beam to the tumor structure by 3-dimensional conformal radiotherapy (3D-CRT), followed by more advanced modalities of beam modification such as intensity-modulated RT (IMRT) and IMRT-based techniques such as volumetric-modulated arc therapy, tomotherapy, image-guided RT (IGRT) or 4-dimensional RT considering patient motion [28]. For IMRT techniques, beam shaping by alteration of the photon fluence during irradiation and beam delivery from multiple gantry angles improves local tumor control and reduces acute toxicities by a more complex and favorable dose distribution with a marked reduction of high doses to organs at risk. However, IMRT techniques expose a larger proportion of the normal tissue to considerably low doses, also regarded as a risk factor for EBRT-related SPMs, which is assumed to be approximately doubled for IMRT compared to 3D-CRT [29,30,31]. But clinical data are not yet available due to the short duration of clinical application of IMRT. High-precision EBRT of small tumor volumes with high ablative photon doses in hypofractionated regimes with optimized sparing of the normal tissue is achieved by stereotactic body RT (SBRT). SBRT is applied to treat small, early-stage local lung cancer and pancreatic cancer, or metastatic lesions in the brain, bones, lung, or liver. Particle therapy with protons or carbon ions represents another option for precise dose delivery to the tumor while sparing the healthy tissue due to the inverted depth-dose profile of charged particles compared to photons [32]. For charged particles, lower doses are deposited in the entry channel of the beam penetrating the healthy tissue reaching the maximum in the so-called (spread-out) Bragg peak when the particle stops, followed by a steep dose drop that allows sparing of organs at risk beyond the tumor volume [33]. While the relative biological effectiveness of photons and protons is largely comparable, carbon ions show a higher biological impact and thus efficacy in inactivating tumor cells. However, due to the limited availability and high costs of this treatment option, only about 1% of all tumor patients receive EBRT with charged particles [34]. Since proton therapy significantly reduces acute and late toxicities in healthy tissue, it is highly recommended in many clinical scenarios for the treatment of solid childhood cancers [35]. This is particularly true for brain tumors, where proton therapy can reduce side effects such as impairment of neurocognition, hearing, and neuroendocrine functions compared to conventional photon EBRT [36]. However, when not applied as a scanned pencil beam, secondary neutrons produced during the passive scattering of proton beams are discussed as a risk factor for SPMs in this irradiation modality [37]. Overall, IMRT and particle therapies are expected to reduce the risk of SPMs but clinical studies and epidemiological investigations will be available only decades after treatment and high-quality clinical research in this area is highly warranted [38,39,40,41].

### 2.2. Chemotherapy

A large variety of cytostatic drugs has been developed over the past seven decades and is administered to tumor patients in different combinations and multimodal therapy settings to eradicate tumor cells. Commonly used chemotherapeutics are classified according to their mechanism of action into the following five groups: (i) Alkylating agents like nitrogen mustards or platinum-based agents induce DNA inter- or intra-strand cross-links or transfer alkyl groups to the guanine residues of DNA resulting in mispair formation in DNA bases and prevent strand separation during DNA synthesis. (ii) Antimetabolites like 5-fluorouracil interfere with essential biosynthetic pathways, disturb the synthesis of DNA and RNA, or cause the formation of DNA strand breaks through inhibition of enzymes like ribonucleotide reductase and DNA polymerase or promote the incorporation of false structural base analogs into the DNA. (iii) Topoisomerase inhibitors like topotecan or doxorubicin inhibit the DNA-processing activity of these enzymes causing DNA strand breaks. (iv) Mitotic spindle inhibitors such as taxanes or alkaloids modify the function or formation of spindle microtubules and thereby inhibit the segregation of chromosomes and nuclear division causing a mitotic arrest and finally cell death. (v) Other chemotherapeutic agents including enzymes, proteasome inhibitors, tyrosine kinase inhibitors, and antibiotics with specific and non-homogenous mechanisms of action [42].

The introduction of multiagent CT helped to reduce the application of high-dose EBRT as the highest risk factor for sequelae including SPMs, in particular for childhood cancer patients. However, also the administration of cytostatic drugs is associated with dose-dependent adverse health effects [43]. Besides the well-known acute effects such as nausea and vomiting, long-term consequences occur primarily in proliferating tissues, e.g., in the hematopoietic system as myelo- and immunosuppression, the gastrointestinal tract, the reproductive system or as pneumopathies and cardiovascular diseases, neurotoxicity, mucositis, and nephro- or hepatotoxicity [44]. One of the most concerning side effects of CT is cardiotoxicity which can vary from subclinical myocardial dysfunction to irreversible and often fatal heart failure [45]. It is induced in a dose-dependent manner primarily by anthracyclines which are widely used for therapeutic intervention in probably more than 50% of all childhood cancer patients posing them with an elevated risk for cardiomyopathies in their later life [46].

Apart from dose-limiting non-carcinogenic normal tissue toxicities, cytotoxic drugs also represent a serious risk for SPMs. CT-related SPMs are primarily a sequela of alkylating agents and the epipodophyllotoxin etoposide with AML occurring most frequently besides ALL, chronic myelogenous leukemia (CML), and myelodysplastic syndrome (MDS) [47]. A higher incidence of CT-related second primary AML is seen from 2–4 years after the start of treatment peaking after 5–9 years. CT-induced leukemias are highly therapy-resistant with cure rates reaching only 10–20% and different subtypes are characterized by genetic alterations [48]. Alkylating agent-related leukemias demonstrate deletions on chromosomes 5 or 7 and topoisomerase-inhibitors like epipodophyllotoxin can induce oncogenic 11q23 translocations involving the MLL gene, the latter dominating in younger patients with shorter latency for leukemia [49]. Overall, high doses of alkylating agents elevate the risk of leukemia as an SPM by a factor of 5–24, depending on the dose, which might be further increased by combination with doxorubicin [50]. Second primary AML has been observed in up to 25% of patients with HL after EBRT plus multiagent MOPP (mustargen [mechlorethamine], oncovin [vincristine], procarbazine, and prednisone) CT [4] or after treatment with the phenylalanine derivative melphalan in patients with multiple myeloma, ovarian carcinoma, or breast cancer.

Besides leukemia, other SPMs are common after CT including NHL or bladder cancer, the latter most frequently observed after treatment with cyclophosphamide [50]. Also, combining CT with EBRT for the treatment of childhood cancer can significantly increase the risk of various solid SPMs. In particular, the use of alkylators and anthracyclines with concurrent EBRT elevates the risk for second primary breast [51], lung [52], stomach [53,54], pancreas [55], thyroid [56,57], or colorectal cancer [58] and sarcomas [59,60,61]. The risk of SPMs for combined treatments with EBRT and CT depends not only on the mode of action but also on other factors such as the tissue involved. For the treatment of HL with EBRT and cyclophosphamide, the risk for bladder cancer is additive [62] but shows synergistic effects and multiplicative risk for gastrointestinal cancer [63]. Overall, these clastogens show differences in the mechanisms of action for carcinogenesis and the associated latencies for SPM. e.g., therapy-related AML triggered by the aforementioned 11:23 translocations induced by topoisomerase inhibitors have much shorter latencies compared to alkylating CT- or EBRT-related AML which requires the acquisition of genomic instability and multiple subsequent genetic alterations for malignant transformation.

### 2.3. Targeted and Immune-Based Therapies

Besides EBRT and CT, approaches that target cancer-selective molecular and immunologic characteristics have made their way into the clinic, also for the treatment of pediatric tumors. In addition to a therapeutic benefit and reduction of morbidity, molecularly-targeted and pathway-directed treatments, as well as immunologic therapies, may provide the greatest impact for childhood cancer patients due to their non-genotoxic mode of action and the reduction of risks for severe late effects including SPMs.

Screening and identification of molecular alterations as well as immune-profiling of pediatric tumors helped to facilitate more accurate patient stratification and personalize combination therapy to overcome resistance to CT and EBRT, achieve optimal treatment outcome, and minimize iatrogenic adverse effects. In the era of precision medicine, it is now also feasible to analyze tissue from pediatric solid tumors or liquid biopsies for genetic aberrations promptly to identify specific targets and adapt and individualize clinical strategies [64,65]. However, compared to adult cancer patients, the use of targeted small-molecule therapeutics and immunotherapies in pediatric oncology is still very limited due to the challenging rarity of cases, difficult-to-drug target structures, and the need for pediatric formulations. In general, childhood cancers are very distinct from adult cancers in terms of cellular origins, genetic complexity, driver mutations, and underlying mutational processes not allowing for a general extrapolation of treatment guidelines from adults to children [66,67,68].

The vast majority of targeted therapies are based on molecular interference with the hallmarks of cancer [69]. The most efficient target structures as oncogenic drivers have proven to be tyrosine receptor kinases (TRKs) involved in cell growth and proliferation including ALK, FGFR, NTRK, PDGFR, EGFR, KIT, and MET or the RAS-MAPK and PI3K-AKT-mTOR signaling pathways showing a high degree of overlap and redundancy offering the possibility for combinatorial treatments [66]. The two major types of targeted strategies use monoclonal antibodies which block the function of cell surface receptors or small molecules like TRK inhibitors [70]. Cell cycle regulators (CDK4/6, CDKN2A, CDKN2B, Wee1, CHK1) and components of the DNA repair machinery (PARP, DNA-PK) are also frequently dysregulated in many tumors and represent important targets for small molecule inhibitors, also to increase the sensitivity towards genotoxic CT and EBRT [71]. Besides, different pediatric tumors show specific alterations of signaling pathways providing potential targets for molecular strategies which will be discussed in the respective sections for each tumor entity.

Cancer immunotherapies manipulate the host immune system to reactivate the anti-tumor immune response and to overcome cancer immune escape. Starting with the application of various cytokines like IL-2 or IFN α-2b, a variety of immunologic anti-cancer strategies have been developed such as the use of adoptive T cell therapy, oncolytic viruses, cancer vaccines, and the use of immune checkpoint inhibitors (ICI) as monoclonal antibodies targeting CTLA-4 and PD-1 or its ligand PD-L1 as the most important development in cancer therapy during the past decade. For a detailed overview of the mechanisms of action of immune-based therapies as applied for pediatric tumor patients, we refer to a recent review by Mackall et al. [72].

Despite the aforementioned benefits, targeted therapies and immunologic strategies are also fraught with side effects and even life-threatening toxicities which require special caution, early detection, and initiation of age-appropriate countermeasures in children and adolescents [73]. For molecular targeted therapies, the majority of adverse effects are of low to moderate severity and predictable on-target toxicities of the inhibited signaling pathway but life-threatening complications may also occur [74]. The most common severe side effects are related to the application of anti-angiogenic agents like monoclonal anti-VEGF antibodies (bevacizumab) causing gastrointestinal and cardiovascular complications. Targeting the receptor kinases EGFR (erlotinib, gefitinib, and cetuximab) and HER2 (trastuzumab) has been mainly associated with respiratory distress and dysfunction, cardiomyopathy, or hypersensitivity events that require medical intervention and countermeasures.

To date, there is no evidence of an increased risk of SPMs for targeted therapies except for easily manageable squamous cell carcinomas of the skin when treating melanoma patients with BRAF inhibitors (vemurafenib, dabrafenib) [75]. Adverse events related to immunologic strategies are triggered by multiple pathways including autoreactive T cells, autoantibodies, and cytokine release, and most commonly involve the gastrointestinal tract, endocrine glands, skin, and liver and necessitate close and extended monitoring [76,77,78]. Therapies with high doses of cytokines such as IL-2 affect T cells and natural killer cells, which can lead to capillary leakage and a sepsis-like syndrome that, in the worst case, causes organ failure. Toxicities related to CAR-T cell therapies in hematologic malignancies are cytokine release syndrome causing fever to multiorgan failure and immune effector cell-associated neurotoxicity syndrome, also known as CAR-T cell-related encephalopathy syndrome, ranging from disorientation to life-threatening cerebral edema. Management of this toxicity profile is usually performed using IL-6 antagonists (tocilizumab, siltuximab) or corticosteroids (dexamethasone). Immune checkpoint inhibitors unleash the antitumor activity of T cells thereby increasing the probability for organ-specific inflammatory responses and immune-related adverse events. The use of anti-CTLA-4 monoclonal antibodies has been associated with adverse events like colitis and hypophysitis whereas anti-PD-1 treatment causes mainly pneumonitis and thyroiditis [79]. Countermeasures for ICI-related toxicities include glucocorticoid therapy with methylprednisolone, hormone therapy, and immune suppression.

So far, experience has shown that immunotherapies in principle do not pose a risk for SPMs. However, e.g., there is a theoretical risk of insertional mutagenesis for CAR-T cell therapy with lentiviral and retroviral vectors [80] but hitherto no case of SPM has been reported when using replication-competent retroviruses or lentiviruses [81]. Rather, CD19-targeted CAR-T cell therapy has been associated with lineage switching from a primary B-ALL to AML and vice versa as a mechanism of immune escape and relapse [82]. Nevertheless, long-term follow-up with close prospective monitoring for SPMs is important and highly recommended also for immunotherapies [83,84] often applied in multimodal oncologic settings where genotoxic cancer therapies induce immunogenic cell death and stimulate cancer immunity to increase the often very low response rates [85,86].

## 3. Childhood Cancer and Second Primary Malignancies

**Figure 1:**
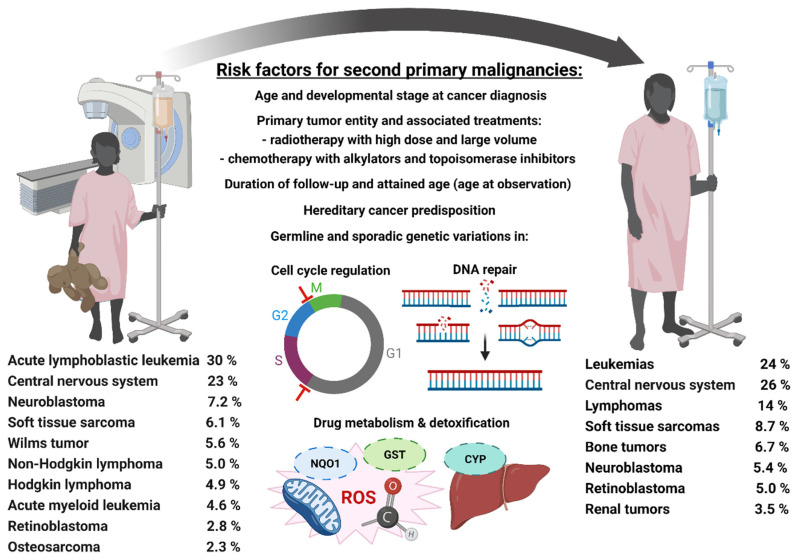
Pediatric first primary malignancies and risk factors for the development of second primary malignancies. The fractions of the most common pediatric first primary malignancies and second primary malignancies in childhood cancer survivors among all observed cancers are provided [11,88,89]. *Abbreviations*: DNA, deoxyribonucleic acid; NQO1 Nicotinamide adenine dinucleotide phosphate: quinone oxidoreductase; GST, Glutathione s-transferase; CYP, Cytochrome P450.

Approximately 200,000 children and adolescents are diagnosed with cancer every year worldwide [87]. Among the most abundant pediatric primary tumors occurring under 15 years of age are ALL (30%), tumors of the brain and central nervous system (CNS, 23%), NB (7.2%), soft tissue sarcoma (STS, 6.1%), Wilms tumor (5.6%), NHL (5.0%), HL (4.9%), AML (4.6%), RB (2.8%) and osteosarcoma (OS, 2.3%) [11,88]. A survey of the most common pediatric first primary malignancies as well as SPMs in childhood cancer survivors and risk factors for their development is provided in Figure 1.

Pediatric cancers reflect a very heterogeneous group of disorders of mostly unidentified etiology and only 5–10% of early-onset malignancies can be ascribed to known inherited or de novo familial mutations in high-penetrance predisposing genes [66,90,91,92,93,94]. Cancer predisposition syndromes include Li–Fraumeni syndrome (LFS) with mutant TP53 which is associated with various benign and malignant neoplasms, including STS, OS, brain tumors, NB, and other cancers [95]. Mutations of RB1 are associated with RB development, but also highly predispose to SPMs such as OSs. Mutations in SUFU or PTCH1 in Gorlin syndrome are strongly associated with the development of medulloblastoma (MB) of the SHH subgroup [91]. Mutations in NF1 can cause neurofibromas (NF), low- and high-grade gliomas (L/HGGs), and malignant peripheral nerve sheath tumors. Mutations in DICER1 are related to proneness to pleuropulmonary blastoma, kidney tumors, CNS tumors, and embryonal rhabdomyosarcoma (RMS) [91]. SMARCB1 or SMARCA4 mutations predispose to renal and extrarenal rhabdoid tumors, meningioma, and small-cell carcinoma of the ovary [96].

Rare syndromes of impaired DNA repair and chromosomal instability like ataxia-telangiectasia (AT), Nijmegen breakage syndrome (NBS), Werner syndrome (WRN), Bloom syndrome (BLM), and Fanconi anemia (FA) highly predispose children to various tumors and, fatally, cause normal tissue hypersensitivity towards genotoxic cancer therapies [91,97]. Genetic predisposition in a subset of pediatric cancers has been related to certain characteristics like (i) familial history of the same or related cancers, (ii) bilateral, multifocal, or multiple cancers, (iii) earlier age at diagnosis than sporadic tumors of the same type, (iv) physical findings suggestive of a predisposition syndrome; and (v) occurrence of specific tumor types that frequently occur in the context of genetic predisposition [98]. However, family history does not seem to necessarily predict the presence of an underlying predisposition syndrome in most pediatric and adolescent cancer patients [92]. Hereditary predispositions are most frequently observed for adrenocortical carcinomas with the most common TP53 germline mutations in LFS (50%) and B-ALL (28%), followed by K27wt HGGs, RMS, MB, and RB (15–25% each). Most germline variations are related to DNA repair genes from mismatch (MSH2, MSH6, PMS2) and double-strand break (TP53, BRCA2, CHEK2) repair [90]. Carriers of such germline mutations are at an about 50% risk for early-onset cancer compared to 1% overall and are prone to iatrogenic SPMs [99,100,101,102]. In general, aggressive tumors with more pronounced genomic instability and chromothripsis are related to a ‘BRCAness’ phenotype due to BRCA1/2 or PALB2 inactivating germline mutations or to TP53 mutations [90,92]. TP53 is also the most common somatically mutated gene in about 4% of childhood cancers, followed by KRAS, the chromatin remodeler ATRX, NF1, and RB1 in 1–2% of pediatric tumors [90]. Albeit mutation frequencies in primary pediatric tumors ranging between 0.02–0.49 mutations per megabase for different tumor entities are generally 14 times lower than in adult cancers, approximately 50% of primary childhood cancers carry a potentially targetable genetic event [90]. This highly warrants further in-depth exploration and clinical trials for individual precision medicine with targeted therapies as well as for predictive biomarkers to improve survival rates while reducing treatment-associated sequelae including SPMs [66].

Although inheritance of rare gene variants associated with pathogenic human cancer susceptibility syndromes can predispose to SPMs, they contribute only a minor fraction to the risk of SPMs at the population level. General and established risk factors for SPMs are age at the initial treatment of the primary malignancy and attained age (age at the observation of an SPM), with young cancer patients at the highest risk for SPMs with increasing age after therapy [103]. In addition, the diagnosis of the primary tumor also plays a crucial role, and the associated treatments with RT or CT and their risks of SPMs as described in Section 2.1 and Section 2.2 It is also commonly assumed that genetic alterations in DNA repair and damage response pathways increase the inherent vulnerability to adverse side-effects of oncologic therapies but the major causal factors for the general population of pediatric cancer patients remain to be unraveled [104,105,106,107]. On the one hand, loci that determine a higher proneness to SPMs might be related to dose modifying proteins which are involved in cytostatic drug metabolism including uptake, activation, and detoxification, or which exert antioxidative properties and reduce the level of indirect IR-induced DNA damage [108]. Such loci may include GSTs, NQO1, cytochrome P450, or TPMT [108]. On the other, loci and pathways may be affected that modify the cellular response to DNA damage and downstream signaling cascades, e.g., XPD for nucleotide excision repair, MSH2 for DNA mismatch repair, XRCC1 for DNA single-strand repair, or Rad51 for homologous recombination [108]. Of note, the risk of SPMs in childhood cancer survivors who are at an elevated cancer risk per se may be polygenic, i.e., the cumulative risk is determined by coinheritance of putative ‘risk’ alleles at several loci [108,109].

Among the most frequent SPMs observed after cancer treatment during childhood or adolescence are leukemias (23.5%), CNS tumors (26.3%), lymphomas (13.5%), STS (8.7%), bone tumors (6.7%), RB (5.0%) or renal tumors (3.5%), often with dismal prognoses [89,110,111,112]. After primary cancer during childhood or adolescence, cumulative incidences of SPMs reach about 12% at 20 years after the diagnosis of the primary tumor [113,114,115,116,117,118]. According to data collected by the German Childhood Cancer Registry (GCCR) from 1980 to 2018, there is a cumulative incidence of 6.8% to develop an SPM within 30 years after the diagnosis of a primary childhood tumor [119]. Thus, about one out of 150 childhood cancer survivors will develop an SPM 30 years after diagnosis of the primary pediatric malignancy. In the following section, we discuss the most abundant primary childhood tumors, their epidemiology and etiology, past, current, and potential future treatments as well as associated risks for late effects including SPMs. Also, the role of these tumor entities as SPMs after treatment of a pediatric primary malignancy is considered. We provide overviews for pediatric hematologic tumors, brain tumors, sarcomas as well as other tumors and the risk factors for their development, current treatments, most common non-cancerous late effects and SPMs, as well as risk factors for their development in Table 1, Table 2, Table 3 and Table 4, respectively.

### 3.1. Hematologic Malignancies

Table 1 provides general information on the most relevant pediatric hematologic tumors, risk factors for their development, current treatments, the most common non-cancerous late effects and SPMs as well as risk factors for their formation.

#### 3.1.1. Acute Leukemia

##### Acute Lymphoblastic Leukemia

Leukemia is the most common cancer diagnosis in children who are younger than 15 years, with an overall incidence of 4.3 per 100,000 in the US and representing 25% of all childhood cancers with ALL accounting for about 76% of all new childhood leukemia diagnoses [9]. Known risk factors for childhood ALL are IR and certain genetic disorders [172,173]. The etiology of ALL is mainly based on cytogenetic alterations including germ-line and somatic karyotypic abnormalities, translocations, and deletions. Germ-line abnormalities associated with childhood leukemia include Down syndrome (DS), FA, Klinefelter syndrome, and AT [120,121]. Somatic alterations include numerical changes such as aneuploidy, pseudodiploidy, and hyperdiploidy [174]. Translocations are frequently present in pediatric ALL including the ETV6-RUNX1 (t(12;21)(p13;q22)) chromosomal translocation [123], KMT2A translocations (t(4;11)(q21;q23)) for infant and therapy-related leukemia [175], and other translocations or deletions frequently involving chromosomes 1, 4, 6–9, 11, 12, 14, 19, 21, and 22 [122].

Over the past 50 years, the therapeutic progress for the treatment of pediatric ALL increased the survival rates from less than 10% in 1962 to about 90% in 2009 [176]. This success is primarily attributed to CT as first-line treatment, and, to some extent, to allogeneic hematopoietic stem cell transplantation (HSCT). CT is generally based on the Assoziazione Italiana Ematologica Oncologia Pediatrica-Berlin Frankfurt Muenster (AIEOP-BFM) ALL protocols [124,125]. Upfront treatment with various cycles of intense multiagent CT includes prednisone, cyclophosphamide, cytarabine, dexamethasone, etoposide, ifosfamide, methotrexate, doxorubicin, mercaptopurine, vincristine, and intrathecal therapy [126]. Advanced stages of the disease are treated with additional doses of vincristine and methotrexate. CNS involvement or prophylaxis requires the intrathecal administration of CT, systemic administration of CT able to penetrate the blood-brain barrier, and/or cranial or craniospinal EBRT [127]. This treatment and particularly EBRT is associated with detrimental effects on growth, neurocognition, and a 22-fold excess risk of second primary CNS tumors that are observed in up to 90% of patients who received cranial EBRT [131,132,133,134,135]. The risk for brain tumors is dependent on radiation dose and is highest for patients treated under the age of 5 years [134]. For all SPM entities that develop with a latency of 9.2 years (range: 0.5–31.7) after diagnosis, survivors of childhood ALL display a high and about 14-fold elevated overall risk compared to the general population with cumulative incidences of 4.2% at 15 years and up to 10.9% at 30 years after diagnosis [136,177]. As the risk of SPMs does not seem to level off even decades after the primary cancer diagnosis, there is a considerable prolonged or even lifelong risk for therapy-associated SPMs in survivors of a primary ALL in childhood [136]. Besides the most common SPMs in survivors of childhood ALL occurring as meningioma, MB, or glioblastoma, these patients develop subsequent primary leukemia or lymphoma, thyroid, breast and bone cancer as well as STS or squamous cell carcinoma of the skin, mainly non-melanoma skin cancer [12,133,136]. In addition to cranial EBRT, the development of other solid SPMs is associated with the administration of alkylating cytostatic drugs (cyclophosphamide) and the emergence of myeloid SPMs due to topoisomerase II inhibitors (epipodophyllotoxins) promoting chromosomal translocations but also by high-dose therapy with methotrexate and mercaptopurine [135].

The 10-year cumulative incidence of death related to any SPM in childhood ALL survivors is 41.1 ± 2.1% and encouragingly the 10-year probability of survival showed a constant increase from 18.9 ± 6.9% for patients with SPMs before 1990 up to 40.9 ± 6.3% for patients diagnosed with an SPM after 2000 [135]. For the different SPM entities that increased morbidity rates in childhood ALL survivors, AML was associated with 5-year survival estimates of 11.2 ± 2.9% before 2000 and 34.1 ± 6.3% after 2000, MDS was associated with 5-year survival estimates of 17.1 ± 6.4% before 2000 and 48.2 ± 10.6% after 2000 and non-meningioma brain tumors showed constantly poor 5-year survival estimates of 19.6 ± 5.5% before 2000 and 16.6 ± 5.3% thereafter [135]. Although patients with squamous cell carcinomas as SPMs showed a high 5-year survival of 71.4 ± 17.1%, all other carcinomas (breast, gastrointestinal, liver, peritoneal, pancreas, lung, cervix uteri, urinary tract, and nasopharyngeal) reached just 40.1 ± 13.7% at 5 years and even dropped to 0% at 10 years. Patients with second primary NHL who did not receive a prior HSCT during ALL treatment show a 5-year survival of about 70% [135].

Recent advantages in the treatment of patients with relapsed/refractory (R/R) ALL have been achieved through low-toxicity targeted therapies with molecular therapeutics and immunologic strategies. Leukemic cells at diagnosis *versus* relapse show significant molecular and clinical differences including *de novo* chromosome aberrations and mutations as well as adaptive resistance to CT [178]. E.g., the expression of the surface antigens CD20, CD19, and CD22 in more than 90% of leukemic blasts can serve as targets for monoclonal antibody therapy in B-ALL [128]. Improved response rates and outcomes in children with B-ALL in the R/R setting were achieved by the approval of monoclonal antibody-based drugs such as blinatumomab, a bispecific T-cell engager targeting CD19 and T-cell receptor-CD3 complex in 2014 and inotuzumab ozogamicin, a cytotoxic anti-CD22-calicheamicin conjugate in 2017 as well as anti-CD22 or -CD19 CAR-T cell-based therapies, e.g., with tisagenlecleucel in 2017 [129]. Another promising therapeutic approach is targeting specific molecular structures or signaling cascades in leukemic cells, e.g., TRK signaling pathways that can be constitutively activated through genetic alterations, paradigmatically shown for the BCR-ABL1 fusion protein produced by the Philadelphia chromosome [130]. The administration of small-molecule TRK inhibitors such as dasatinib, ruxolitinib, or crizotinib improved prognosis when included in ALL treatment regimens [130]. These therapeutic strategies are successful approaches to achieve durable remission and to de-intensify conventional CT thereby reducing unacceptable toxicity, mortality, and serious late complications for patients with R/R disease.

##### Acute Myeloid Leukemia

AML is the second most common pediatric hematopoietic malignancy accounting for 15–20% of childhood leukemia [88] and is highest in infants aged 0–1 years whereas it is a rare event in children up to 18 years [179]. Albeit intensification of standard CT and improvements in risk classification as well as supportive care increased the survival rates for pediatric AML up to 70%, most prognoses remain unfavorable and relapse rates with poor outcomes still reach 50% [140,180,181]. The incidence of acute leukemia is below 3% of all tumors but represents the leading cause of cancer-related death in children and persons younger than 39 years of age [182].

The development of AML is associated with genetic disorders and exposures to viruses, IR, chemicals, or previous CT [137]. Genetic disorders and constitutional genetic defects predisposing to AML are DS, Klinefelter’s syndrome, LFS, FA, and NF [137]. The most frequent karyotypic abnormities involve the loss or deletion of chromosome 5, 7, Y, and 9, translocations such as t(8;21)(q22;q22), t(15;17)(q22;q11), or trisomy 8 and 21 [138]. Recent comprehensive sequencing studies have strongly expanded the spectrum of predisposition genes in families with leukemia, including TP53, RUNX1, IKZF1, and ETV6 [139].

The therapy of AML is very challenging due to high molecular heterogeneity, high relapse, and therapy toxicity. The treatment of AML primarily relies on the BFM studies [183] including four to five courses of intensive myelosuppressive CT, mainly based on anthracyclines and cytarabine with or without HSCT [140,141]. CNS-directed treatment at initial diagnosis of pediatric AML is recommended and implemented as a standard component of most AML therapy protocols as prophylactic intrathecal CT whereas cranial EBRT has been mostly reduced or even omitted [142]. As for ALL, targeted therapy strategies have been developed for AML to increase the therapeutic success with less toxicity but showed only modest or no success. The primary molecular target structure in AML blasts is CD33, based on which the humanized anti-CD33-calicheamicin conjugate gemtuzumab ozogamicin, as well as ineffective therapies of CD33- or CD123-based CAR-T cells, were developed [143,144]. The frequent mutation of the TRK FLT3-ITD in AML led to the clinical implementation of the small molecule TRK inhibitors sorafenib, midostaurin, gilteritinib which are currently tested in clinical trials with newly diagnosed pediatric AML patients carrying an FLT3 mutation [145,146].

Due to the high mortality of pediatric AML patients and only a few long-term survivors, the risk of therapy-related late effects and associated SPMs is rather small and the assessment is complicated. Leung et al. [147] showed a 10-fold greater risk of SPMs in survivors of childhood AML treated at a mean age of 8.7 years during their follow-up for 10.6 ± 6.1 years since the primary diagnosis when compared to the general population. SPMs that were found in five patients out of this cohort of 501 patients were two mucoepidermoid carcinomas, one NHL, one supratentorial primitive neuroectodermal tumor, one ALL and one patient developed a meningioma as a third primary malignancy. However, at 15 years after the diagnosis of the primary AML the estimated cumulative incidence of SPMs (1.34 ± 0.61%) was outdone by the high cumulative incidence of death due to any other cause (72.96 ± 2.14%).

##### Acute Leukemia as Second Primary Malignancy

The development of second primary acute leukemia is among the most detrimental late-effects of cancer treatments. Secondary primary leukemias represent about 20% of all SPMs and occur as second primary ALLs or more frequently, perhaps exclusively, as second primary AMLs [60,174,184,185]. The cumulative incidence of treatment-related AML is approximately 0.6% at 15 years after diagnosis in children treated for ALL or solid tumors [114,186]. The treatment of systemic and solid cancers like HL, breast cancer, head and neck cancer, bone cancer, and sarcoma by EBRT and CT are well-known risk factors for subsequent primary myeloid malignancies [187,188], in particular the application of alkylating agents and topoisomerase II inhibitors. The risk for AML after intense CT is highest for STS, OS, testicular cancer, anal cancer, and brain tumors [187] and is potentially increased up to 100-fold [189]. Also, EBRT alone can increase the risk for second primary AML and MDS, but since the advent of more precise EBRT techniques, causal relationships are not that clear anymore [190,191]. The development of second primary acute leukemia shows a maximum within 5–10 years after therapy of the primary malignancy, often with very poor prognosis and outcome. Second primary AML or MDS are often characterized by specific genetic and cytogenetic alterations induced by cytostatic agents, presenting the same chromosome aberrations and gene mutations as *de novo* entities [189]. Deletions or loss of 7q or monosomy 7 with normal chromosome 5 and deletions or loss of 5q or monosomy 5 are closely related to previous therapy with alkylating agents [192]. Topoisomerase II inhibitors are well-known inducers of balanced translocations involving chromosome bands 11q23 (MLL) or 21q22 (RUNX1) primarily in children, mostly representing the sole karyotypic change in therapy-related AML with a short latency period of 1–5 years [193]. The application of topoisomerase II inhibitors, anthracyclines, mitoxantrone [194] and EBRT [195] has also been related to therapy-associated acute prolymphocytic leukemia with various other translocations and chimeric rearrangements [196]. In general, the most prominent rearrangements include MLL at 11q23, AML1 at 21q22, RARA at 17q21, and CBFB at 16q22, leading to a dominant loss-of-function of transcription factors and impairment of differentiation [193].

#### 3.1.2. Non-Hodgkin Lymphoma

NHL is a heterogeneous group of lymphoid malignancies with multiple subtypes, each of which has distinct morphologic, immunophenotypic, and clinical features [197]. It is the fourth most common malignancy in children accounting for about 7% of all cancers in patients under 20 years of age with a higher incidence in adolescents [198,199,200]. The majority of childhood NHLs are high-grade Burkitt’s lymphomas, mainly of B-cell origin, and less than 5% represent other types such as peripheral T-cell lymphoma, extranodal natural killer/T-cell lymphoma, and follicular lymphoma whereas adolescents show a higher prevalence of large-cell lymphomas [201,202]. Children usually develop an extranodal disease involving the mediastinum, abdomen, and/or head and neck, as well as the bone marrow or CNS [198]. The general etiology of NHL is poorly understood, with the only established risk factors being infection and immune dysregulation [148].

A predisposition to NHL has been associated with various auto-immune diseases or infections which, however, are relatively uncommon in the general population and can justify only a minor fraction of NHL cases. Although some studies have identified genetic risk factors for NHL, including polymorphisms in the TNF and IL10 cytokine pathways, toll-like receptor or DNA repair genes including RAG1, LIG4, ERCC5, WRN, MGMT, and XRCC1, such findings are not yet conclusive [148]. The treatment of childhood NHL is mainly based on the three consecutive multicenter study protocols NHL-BFM 86, 90, and 95 and their further development [149,150,203,204,205,206]. After the NHL-BFM 95 study, NHL patients were stratified according to morphological, genetic, and immunological aspects into lymphoblastic lymphomas, mature B-cell lymphomas, or anaplastic large cell lymphoma to adapt cumulative doses and intensity of CT to the aggressiveness of the disease contrary to the previous ‘one-size fits all’ approach. EBRT for NHL has been largely reduced or omitted already during the 1970s in the pediatric NHL-BFM-trials and is mainly applied as cranial EBRT if the CNS is involved in addition to CNS prophylaxis with intrathecal CT and methotrexate [206]. The addition of immunotherapeutic agents further improved cure rates and survival of patients in advanced stages of NHL. With this constant improvement of NHL treatment, the 5-year survival rate increased from 45% to 87% in children younger than 15 years and from 48% to 82% for adolescents aged 15–19 years between 1975 and 2010 [207].

Long-term survivors of NHL are at a generally lower risk for late effects and mortality than observed in survivors of other primary childhood tumors with a risk for all-cause mortality (standardized mortality ratio, SMR) of 4.2 and, notably, increased risk of death from SPMs (SMR: 26.7), cardiac disease (SMR: 6.9) and pneumonia (SMR: 15.4) [157]. Again, these adverse late effects have been attributed to high cumulative doses of alkylators, topoisomerase II inhibitors, and anthracyclines [158]. The most frequent SPMs in survivors of childhood NHL are carcinomas followed by AML and lymphoid malignancies with an overall cumulative incidence of about 5.7% at 20 years and a median latency of 8.7 years [12,158,208]. There are several known risk factors for the development of SPMs after pediatric NHL including sex (female), lymphoblastic lymphoma, CNS involvement, and cancer-predisposing syndromes like AT, NBS, and constitutional mismatch repair deficiency [158]. The occurrence of NHL as an SPM in former childhood cancer patients is a rare event. According to the NHL-BFM study center, only 0.3% (11/2.968) of the total newly diagnosed NHLs after a previous malignancy other than NHL were proven to be a subsequent SPM [209].

Promising perspectives for therapeutic improvements are the implementation of novel and mainly immunologic therapies for NHL treatment, in particular for the pediatric R/R setting of NHL with very poor survival rates ranging from 27–36% [151,152,153]. Adding rituximab, a chimeric murine/human monoclonal antibody with a high affinity against CD20 on B cells inducing complement-mediated lysis and antibody-dependent cell-mediated cytotoxicity to CT, even to substitute EBRT, showed marked prolonged event-free overall survival among children and adolescents with high-grade, high-risk NHL and may help to reduce the risk of cardiotoxicity and SPMs [154,155]. Other promising therapeutic approaches in pediatric and adult NHL in the R/R setting are antibody-drug conjugates (inotuzumab-ozogamicin, polatuzumab-vedotin, pinatuzumab-vedotin), CAR T-cell therapy (tisagenlecleucel), or strongly NHL subtype-dependent therapies targeting BTK (ibrutinib) [156]. Alternative strategies of oncologic therapies that have only been tested in clinical trials with adult NHL in the R/R setting include bispecific T-cell-engaging antibody constructs (blinatumomab, mosunetuzumab), specific therapies targeting the antiapoptotic protein BCL2 (venetoclax), the E3 ubiquitin ligase cereblon (lenalidomide) or the NF-κB pathway (bortezomib) as well as ICI against PD-1 (nivolumab, pembrolizumab) [156]. However, despite this promising outlook, the transfer of beneficial study outcomes from adult to pediatric NHL patients remains challenging due to large molecular differences and the general heterogeneity of this disease.

#### 3.1.3. Hodgkin Lymphoma

Classical HL represents about 5–6% of all childhood cancers and 10% of all lymphomas with an incidence rate of 12 cases/million/year in the 0–14-year age group with male predominance. The incidence is higher in adolescents and young adults accounting for 15% of cancer diagnoses in this 15–24-year age group or adults around 59 years of age [10,210]. HL is grouped into four histological subtypes: nodular sclerosis, mixed cellularity, lymphocyte rich, and lymphocyte depleted [211]. All forms of HL are characterized by the occurrence of pathognomonic multinucleated Reed-Sternberg cells, loss of the B-cell markers CD19 and CD20, expression of CD15 and CD30, and the presence of Epstein-Barr-Virus positive and negative forms [159]. The etiology of HL in early life is heterogeneous and still poorly understood. Besides the Epstein-Barr-Virus infection in about 20–40% of HL cases, it may involve genetic factors, immune-related disorders, other infections, and environmental exposures [159]. As for NHL, family studies indicate that the risk for HL shows a familial predisposition [160]. In general, the development of primary HL most likely involves a complex interplay between genetic susceptibility, immune impairment, and environmental exposures.

HL diagnosed at an early stage represents the most curable hematologic malignancy with long-term survival rates now exceeding 90% after treatment with CT alone or combined with EBRT [165]. Therefore, patients treated for pediatric HL are at the highest risk for treatment-related sequelae among 5-year survivors of all childhood tumors [12]. Former HL patients have a two- to fourfold increased risk to develop an SPM or cardiovascular disease compared with the general population, representing the leading cause of death in these patients. Other frequently observed non-carcinogenic adverse late-effects are pulmonary dysfunction, endocrinopathies (thyroid dysfunction, infertility), neck muscle atrophy, and persistent fatigue [165]. Therefore, HL was the first tumor entity where primary attention was paid to the reduction of treatment-related long-term consequences.

Standard CT for childhood HL showed very favorable outcomes already in the 1970s but the applied MOPP regimen was soon associated with severe adverse late-effects such as SPMs, gonadal toxicity, and sterility in females. Former HL patients showed very high risks for subsequent primary leukemia related to alkylating agents, NHL, and solid SPMs related to the excessive use of extended-field EBRT, so-called mantle field EBRT. The MOPP regimen was replaced by ABVD (adriamycin [doxorubicin], bleomycin, vinblastine, and dacarbazine), which in turn led to a decrease in leukemia rates due to reduced use of alkylating agents, but still caused normal tissue toxicity, especially cardiopulmonary complications, related to the application of doxorubicin, bleomycin, and EBRT. Solid cancers represent the most frequently observed SPMs in survivors of a primary HL in childhood related to mantle field EBRT [166,168] and mainly comprise thyroid carcinoma and breast cancer, sarcoma, colorectal carcinoma, melanoma, and cervix carcinoma in descending order. A linear correlation between radiation dose and several solid SPMs including lung, breast, stomach, and pancreas cancer has been established [53,55,167,169]. The highest risk factors for SPMs developing after EBRT for HL administered under the age of 10 years are chest exposures for breast cancer in females and lung cancer in males, abdominal/pelvic EBRT with concomitant high-dose alkylating agents for colorectal cancer, and neck exposures for thyroid cancer in females with cumulative incidences by an age of 50 years of 45.3%, 4.2%, 9.5%, and 17.3%, respectively [212]. The risk of breast cancer is primarily attributed to the application of EBRT increasing up to eightfold for more than 25 years post-exposure when doses higher than 40 Gy were administered [213]. With chest irradiation, even lower doses (<20 Gy) have been associated with an increased risk of breast cancer, in particular for large treatment volumes [214]. Alkylating CT can substantially contribute to the risk of solid SPMs in an additive or even synergistic manner with EBRT for lung, stomach, and pancreatic cancer [55,167,170,171]. For patients treated from 1989 through 2000, reduced EBRT volumes, anthracycline-containing CT, lower doses of alkylating agents, and less frequent infra diaphragmatic EBRT was applied compared to previous HL therapy protocols. To date, HLs are treated with involved-field/involved node EBRT and OP/EPA (vincristine, prednisone, procarbazine/etoposide, and doxorubicin) or OPPA/COPP (cyclophosphamide, vincristine, prednisone, and procarbazine) CT. EBRT is usually omitted for patients showing negative positron emission tomography scans after induction CT or an overall good response to CT [161].

Albeit the incidence of SPMs after primary HL in childhood seemed to decrease among 5-year survivors who were treated in the past two decades, primarily because of the declining use of alkylating agent-based CT and reduced volumes and doses of EBRT, survivors of pediatric HL are still at the highest risk for SPMs with no evidence of risk reduction with increasing duration of follow-up. While first studies in children and young adults after low-dose EBRT showed promising results regarding the incidence of SPMs during a median follow-up of 8–13 years [215,216,217], more recent studies with long-term follow-up of 20 years showed a similar cumulative incidence of 17% and latency of 15.4 years for solid SPMs occurring as sarcomas, breast and thyroid carcinomas as found in studies in children with HL who received high-dose EBRT [218]. In general, the cumulative risks for SPMs after treatment of primary HL in childhood range from 7.6% at 20 years up to 21.9% at 25 years [56,115,219,220,221,222,223,224,225,226]. The Late Effects Study Group investigated the occurrence of SPMs in former childhood HL patients diagnosed between 1955 and 1986 at a maximum age of 16 years with a median follow-up of 26.6 years [212,227]. The analyses of this cohort resulted in a cumulative incidence of SPMs of 26.3% at 30 years and 26.4% at 40 years after diagnosis and a 14–18.5-fold increased risk and 6.5–6.7-fold excess risk of developing an SPM for children treated for HL before 1986 compared to the general population. In long-term survivors of pediatric HL treated between 1970 and 1986, the cumulative incidence of SPMs 30 years after the diagnosis of the primary malignancy is as high as 18.4% with an excess risk of 8.7 compared to the general population [12]. The consecutive HL studies conducted in Germany reported a comparable mean cumulative incidence for SPMs of 19% at 30 years after diagnosis of a primary HL in childhood [228]. A recent study by the Netherlands Cancer Institute determined a standardized incidence ratio (SIR) of 4.6 for SPMs at a median follow-up of 19.1 years compared to the general population with a persisting risk for up to 40 years after treatment of adolescent and adult patients at an age between 15 and 50 years and a cumulative incidence for SPMs reaching 48.5% [229]. Pediatric HL patients are also at very high risk for developing multiple subsequent primary malignancies, e.g., with a cumulative incidence of about 21% for third primary malignancies at 10 years after the diagnosis of the SPM as described in the Late Effects Study Group [227].

Therapy concepts beyond conventional EBRT and CT were developed for HL which are mainly applied in R/R settings. R/R is observed in 10–15% of patients with limited-stage disease and 30–40% with advanced stage in all HL patients after a frontline therapy [162]. R/R stages are treated with high-dose platinum- or gemcitabine-based CT and autologous stem cell transplantation with complete response rates ranging between 17–67% [163]. The most prominent approach of alternative therapy strategies in HL is the introduction of the CD30-directed antibody-drug conjugate brentuximab-vedotin or a second-line treatment option as a single agent into salvage protocols and with CT for the treatment of patients with previously untreated stage III or IV disease [230]. Besides CD30 expression, Reed-Sternberg cells show upregulation of PD-L1 and JAK2 related to 9p24.1 amplification [164] building the rationale for the application of ICI directed against PD-1 (pembrolizumab, nivolumab) and the use of JAK2-inhibitors (itacitinib, ruxolitinib). Further strategies include the use of CAR-T cells, histone deacetylases inhibitors (panobinostat), immunomodulatory drugs (lenalidomide), BTK inhibitors (ibrutinib), mTOR inhibitors (everolimus), and CD25-directed antibody-drug conjugates (camidanlumab tesirine) [230].

#### 3.1.4. Hematopoietic Stem Cell Transplantation and Risk of Second Primary Malignancies

Long-term survivors of pediatric hematologic malignancies who received myeloablative HSCT with high-dose CT and fractionated total body EBRT as a conditioning regime are at the utmost risk for severe late-effects and SPMs facing a 10-year cumulative incidence of death of 10.4 ± 1.3% due to treatment-related toxicities and 41.1 ± 2.1% because of SPMs [135]. Children that were younger than 10 years at the time of HSCT show an accumulated 60-fold higher risk for any SPM compared to the general population [231]. In general, HSCT is a treatment option for many hematologic disorders like AML in first remission, ALL with poor prognosis, for chronic myeloid leukemia (CML) besides the TRK inhibitor imatinib (Gleevec^®^) or myeloproliferative disorders other than CML, MDS, chronic lymphocytic leukemia, HL in the R/R setting, myeloma, AL-amyloidosis, acquired severe aplastic anemia or constitutional severe aplastic anemia in FA [232].

If applicable, CT regimes administered with total body EBRT before HSCT consist of different combinations of etoposide, cyclophosphamide, melphalan, vincristine, cytosine arabinoside, thiotepa, and fludarabine. Total body EBRT is usually given as anterior-posterior parallel opposed fields in 6 total fractions at 2 per day delivered at a low dose rate with lung shielding [233].

Common non-malignant adverse late-complications observed in these patients are thyroid dysfunction, growth impairment, hypogonadism, pulmonary dysfunction, or cataracts [233]. SPMs related to total body EBRT are categorized as hematologic malignancies occurring mostly as MDS and AML, post-transplant lymphoproliferative disorder, and solid tumors [231]. While second primary hematologic malignancies as a consequence of the treatment for HSCT are very rare in children and occur more frequently in older patients after treatment with alkylating cytostatics and high doses of total body EBRT, solid tumors represent the most common SPMs for pediatric patients [231,234,235]. Compared to the general population, the risk of solid SPMs after HSCT is 33–36.6 fold higher in pediatric patients treated under the age of 10 years, 4.6 times higher for patients treated at the age of 10–29 years, and about normal for patients receiving treatment when 30 years or older [231,236]. Among the most frequent solid SPMs are tumors of the buccal cavity, liver, brain, and CNS, thyroid, bone and connective tissue, salivary gland, and melanomas with cancers of the brain or thyroid most often observed [231,233,236,237].

To lower or avoid the systemic genotoxicity of preconditioning CT and EBRT, alternative strategies for HSCT-conditioning or therapies have been developed, in particular for hypersensitive patients with syndromes of compromised DNA repair such as FA showing an escalated risk for leukemia and bone marrow failure at a young age. For these patients, successful alternative donor HSCT using T cell-depleted grafts without total body EBRT as well as strategies with reduced or fludarabine-based CT showed excellent outcomes [238,239]. Recently, lentiviral-mediated hematopoietic gene therapy showed progressive engraftment of gene-corrected hematopoietic stem cells in non-conditioned FA patients offering a low-toxicity therapeutic option for such life-threatening diseases [240].

### 3.2. Solid Tumors

#### 3.2.1. Brain Tumors

Brain tumors are the most frequent primary solid tumors in children representing about 25% of all pediatric malignancies and are second to the overall cancer incidence only to leukemias [241,242]. The incidence of malignant CNS tumors in children at an age of 0–14 years is about 3.8 per 100,000 and 17% of these tumors are highly aggressive malignant gliomas [243]. A peak incidence is observed from the age of 10–19 years with a male predominance. The etiology of childhood CNS tumors is largely unknown and only about 5% may be explained by genetic predisposition. Except for high doses of IR, no significant contribution of exogenous exposures to environmental risk factors has been established for CNS tumors so far [244]. CNS tumors are classified according to their location as infratentorial, supratentorial, parasellar, or spinal and include MB, ependymoma (EPN), glioblastoma, anaplastic astrocytoma, and diffuse intrinsic pontine glioma (DIPG) [245]. Optimized therapies improved the 5-year survival rates in children under 15 years of age with CNS tumors from 57% in 1977 to 75% in 2007 [99,246] but the successful treatment with high-dose CT and EBRT is associated with severe and fatal long-term effects [247]. Overall, the prognosis for childhood CNS tumors remains poor and mortality rates are still high. An overview of pediatric brain tumors, risk factors for their development, current treatments, non-cancerous late effects as well as the most common SPMs, and risk factors for their occurrence is provided in Table 2.

##### Medulloblastoma

MBs are embryonal brain tumors and the most frequent and aggressive malignant entity of the cerebellum that accounts for 15–20% of all childhood brain cancers [301,302]. Risk factors for the development of pediatric MB in the posterior fossa are certain hereditary cancer predisposition syndromes like Turcot, Gorlin, Rubinstein Taybi, LFS, and FA. MB are grouped into the molecular entities wingless-activated (WNT, 10%), SHH-activated (30%), group 3 (20–25%) that is characterized by amplification of various proto-oncogenes including MYC (16.7%), PVT1 (11.9%), SMARCA4 (10.5%), and OTX2 (7.7%), and group 4 (35–40%) that is characterized by molecular abnormalities associated with chromosome 17 [248,249]. Survival rates range from more than 90% in the low-risk group to only 50% or less in the high to very high-risk group making MB a major cause of cancer-related mortality in children [248].

MB is commonly treated by surgical resection and adjuvant craniospinal EBRT with concomitant vincristine starting approximately 30 days post-resection [250]. Adverse effects of craniospinal EBRT have been documented as hypothyroidism, growth hormone and adrenocorticotropic hormone deficiency, and altered metabolism [252,253]. EBRT is usually followed by CT according to the ‘Packer regimen’ for adult patients consisting of vincristine and cisplatin plus either lomustine or cyclophosphamide as the standard of care for standard-risk patients plus high cumulative doses of cyclophosphamide for high-risk patients [251].

For SPMs in survivors of a pediatric primary MB, the cumulative 10-year incidence has been reported to be about 4.2% with a high mortality rate of approximately 33% [254,255]. Most SPMs occur as highly aggressive malignant glioma, meningioma, or thyroid carcinoma with a median latency of eight years (range 4.3–11.8) in anatomic regions exposed during EBRT. The deleterious effects of CT and craniospinal EBRT on neurocognitive and neuroendocrine function as well as the development of fatal SPMs give a strong rationale for the development of alternative strategies which are mainly based on the demographic, genomic, and epigenetic profiles of the 4 MB subgroups [256]. Blocking of molecular targets of the specific signaling pathways of each MB subtype might result in more effective and less toxic therapy regimes. Based on the high anti-cancer activity of the SHH pathway-inhibitor ACVR1 in preclinical studies, a variety of clinically active small molecule inhibitors were developed including saridegib, erismodegib, or vismodegib [249], the latter showing promising clinical responses in SHH-driven MB [303]. Further strategies include targeting of CDK4/6, c-Met, Wee1, PI3K/mTOR, EZH2, or CHK1/2 or the BET bromodomain pathways which are currently investigated in clinical trials for MB in the R/R setting [248]. Immunological therapies are also being tested, including PD-1 inhibitors (pembrolizumab, nivolumab), monoclonal antibodies against CD40 (APX005M), or PEP-CMV (cytomegalovirus) based vaccine trials for oncolytic viral therapy [248].

- Gliomas

Gliomas are the most frequent CNS tumors arising from glial cells in the brain or spine and represent approximately 60% of all pediatric brain tumors. About 30–50% of pediatric gliomas are considered high-grade malignancies with dismal outcomes and 5-year survival of less than 20% [301,302,304]. According to the World Health Organization criteria, CNS cancers are classified based on histological features into LGGs (grade I and II astrocytomas) and HGG, such as anaplastic astrocytomas (grade III) and glioblastomas (grade IV) with IDH wildtype or IDH mutant, the latter being uncommon in the pediatric population [245]. Recently, specific molecular features have been incorporated into the classification scheme [245]. Ependymoma (EPN) is usually regarded as a separate entity.

##### Ependymoma

Pediatric EPNs represent the third most common childhood tumor of the CNS accounting for 6–12% of all brain tumors in children peaking between the age of 0–4 years with prevalence in males [301,305]. EPNs are of glial origin and are classified according to their three anatomical compartments (supratentorial, posterior fossa, or spinal) and further subdivisions in nine subgroups according to genetics and DNA methylation profiles [306]. 90% of pediatric EPNs occur intracranially, with two-thirds in the posterior fossa and one-third in the supratentorial compartment [260]. Although most EPNs develop sporadically, there might be an association with infections with the SV40 virus [257] and genetic predispositions like NF type 2, Turcot Syndrome B, or LFS [258,259]. EPN subtypes vary significantly concerning clinicopathologic features, molecular characteristics, and lethality [260].

Most EPNs are treated by maximal surgical resection and adjuvant EBRT [260,261,262]. The application of CT in EPN treatment is still controversial due to the high resistance of EPN. Common CT approaches include platinum derivatives, etoposide, cyclophosphamide, vincristine, and methotrexate, but so far, no CT regimen was superior to adjuvant EBRT [264]. The ten-year overall survival in pediatric EPN patients is 64% but the 5-year survival rate for infancy is only 42–55% since these patients are mostly excluded from adjuvant EBRT despite the highest incidence of EPNs in children under 5 years of age [302,307]. Recent approaches treating classic supratentorial EPN by conformal EBRT with rigorously defined target volume definitions and minimal clinical target volume margins achieved improved survival with reduced neurotoxicity in infants similar to those in older children [266]. Also, proton beam EBRT has been applied for EPN treatment to spare the normal brain tissue and showed comparable outcomes to conventional 3D-CRT [263,308] but sporadic brainstem necrosis after infratentorial proton irradiation may occur as a very rare event [309].

Due to the rarity and heterogeneity of this pediatric malignancy, the development of SPMs has only been described in adult spinal cord EPN patients. Here, SPMs occurred as pancreatic cancer, prostate cancer, HL, intracranial meningioma, mucin-producing pulmonary adenocarcinoma, gastric cancer, and astrocytoma 2 months to 20 years after treatment of the primary tumor with a cumulative incidence of 9% [267]. Remarkably, all SPMs developed outside the treatment volume of adjuvant EBRT and none of the patients received CT. This shows an elevated intrinsic risk for SPMs in these patients but still, there is a lack of information regarding the molecular genetics of EPN. Recent studies suggest that a hypermethylated phenotype causing silencing of tumor-suppressor genes like CDKN2A, CDKN2B, HIC1, RASSF1A, CASP8, MGM, and TP73 in EPN may foster the development of second primary CNS tumors [268,269]. However, the genetic landscape of pediatric and adult EPN differs significantly [260]; this also seems to apply to the methylation level [268].

Although targeted therapies appear to have no role in EPN, some strategies are currently being tested in recurrent disease, e.g., inhibition of the TRKs ERBB1 and ERBB2 by lapatinib, interference with the NFκB pathway, or inhibitors of the transcriptional regulator YAP1 [265]. As an alternative strategy, the application of peptide-based vaccines is considered for EPN immunotherapy to improve disease control for high-risk tumors and reduce sequelae for tumors with favorable outcomes [310].

- Low-Grade Gliomas

LGGs represent 40% of all pediatric brain tumors and are mainly pilocytic astrocytomas, which account for 20% of brain tumors in patients under the age of 20 years. Other childhood LGGs include gangliogliomas, subependymal giant-cell astrocytoma, fibrillary/diffuse astrocytoma, pilomyxoid astrocytoma, pleomorphic xanthoastrocytoma, oligodendrogliomas, and oligoastrocytoma. However, the classification of LGGs is challenging and about 30% are commonly categorized as non-specific. The cancer-predisposition syndromes NF1 and tuberous sclerosis complex (TSC1 or TSC2 germline mutation) are associated with higher risks for the development of pilocytic astrocytomas and subependymal giant cell astrocytomas, respectively [270].

The treatment of pediatric LGGs has an excellent outcome with a 20-year overall survival exceeding 90% [276,311]. Childhood LGG is primarily treated by surgery with no adjuvant therapy after complete tumor resection showing very positive prognostic outcomes [271]. If applied, first-line CT for LGGs includes carboplatin and vincristine, TPCV (thioguanine, procarbazine, lomustine, and vincristine), and weekly vinblastine monotherapy. First-line CT is primarily scheduled for young patients to avoid sequela of EBRT occurring as cognitive side effects, vasculopathy, and hormonal imbalances that outweigh the benefits of EBRT. Pediatric LGG patients treated by EBRT have significantly inferior outcomes and elevated late mortality compared to patients without EBRT [276].

LGGs frequently show constitutive activation of the oncogenic Ras-RAF and MAPK-ERK pathway by duplication, fusion, and mutation of the proto-oncogene BRAF or through the loss of function of the NF1 tumor suppressor gene offering options for molecular therapies [312]. Inhibitors of BRAF (vemurafenib, dabrafenib, trametinib) and MEK (selumetinib) that downregulate the Ras/ERK pathway have been incorporated into clinical trials for children with LGGs showing promising preliminary results [272,273] but may cause a paradoxical activation of the Ras/ERK pathway in BRAF fusion-positive tumors in contrast to a downregulation in mutated BRAF proto-oncogene with a change of valine to glutamic acid at codon 600 (BRAF^V600E^) [274]. Since the loss of NF1 also causes hyperactivation of the PI3K-AKT-mTOR pathway, mTOR inhibitors (everolimus) have been used in clinical trials with very positive responses for LGGs in children with tuberous sclerosis [275]. Also, the administration of the VEGF-targeting monoclonal antibody bevacizumab showed marked responses in pediatric optic pathway gliomas [313].

- High-Grade Gliomas

HGGs represent 30% of all pediatric gliomas and are associated with very poor outcomes and 5-year survival rates usually do not exceed 20% due to high drug resistance [243,301,314]. The overall survival at 1, 5, and 10 years of age is 65.4%, 25.2%, and 20.4% for anaplastic astrocytoma and 58.1%, 21.8%, and 18.1% for the most pathologically advanced glioblastoma [243]. Comprehensive studies integrating copy number variations, gene expression, and mutation analyses revealed that genomic alterations in pediatric HGGs overlap with, but are distinct from primary adult glioblastoma [315]. Almost all glioblastomas show compromised TP53, PI3K/TRK, and RB pathways [277,278]. Genetic changes in TP53, CDKN2A, and PIK3CA are common in both adult and pediatric patients whereas PTEN mutations and EGFR amplification are more frequent in adult primary glioblastoma [279,280,281].

The standard of care for pediatric HGGs is radical surgery followed by focal EBRT and CT with temozolomide (TMZ) but treatment protocols remained fairly unchanged since 2005 [282]. EBRT of pediatric HGGs is associated with deleterious late effects on cognitive function, neuroendocrine function, vascular changes leading to increased stroke risk, or SPMs [289,290,291], and must be applied with utmost caution. Concomitant treatment of pediatric HGG with EBRT and TMZ showed conflicting outcomes but has a more attractive toxicity profile compared to preceding treatment regimens [316,317,318,319]. The administration of TMZ or other alkylating cytostatics like carmustine in pediatric HGGs has also been associated with the development of SPMs occurring as hematologic malignancies with a cumulative incidence of 20% at 10 years follow-up when applied as a second-line treatment after first-line multiagent CT [292,293,294,295,296].

HGGs show various molecular alterations and clinicopathological features that serve as molecular targets for antineoplastic strategies. HGGs also harbor BRAF^V600E^ mutations and loss of function of NF1 but at a much lower rate compared to LGGs and these alterations occur particularly in secondary HGGs concomitant with CDKN2A and CDKN2B mutations [283]. In these patients with poor prognosis, positive responses were observed for the administration of BRAF and MEK inhibitors [284]. HGGs with TRK fusions (NTRK1, NTRK2, NTRK3) that occur mainly and in up to 40% of infant tumors show good responses to pan-TRK inhibitors [285]. Other targetable alterations in HGGs are activation of the EGFRvIII mutation, aberrant activation of PDGFR A and B, or FGFR1 mutations, MET oncogene fusions, or the PI3K-AKT pathway [286,287]. Although molecular drugs are available for these individual signaling pathways, combination strategies are more promising due to the high heterogeneity and treatment resistance of this tumor entity. Recently, treatment with the monoclonal anti-PD-1 antibody nivolumab showed a striking clinical and radiological response in case reports of selected HGG patients with germline hypermutation syndromes and mismatch repair deficiency associated with an increased mutational load and predicted neoantigens [288]. But so far, no such positive clinical reports are available for the vast majority of pediatric HGG patients without germline hypermutation syndromes.

- Diffuse Intrinsic Pontine Glioma

DIPGs are the most common and deadliest brainstem cancers in children representing 15–20% of all pediatric CNS tumors peaking at a median age of 6–7 years [297]. About 90% of DIPGs are characterized by pathognomonic point mutations in the histone variants H3F3A (65%) or HIST1H3B (25%) making the affected cells susceptible to acquisition of additional mutations, e.g., in TP53 or ACVR1 [297].

Due to the localization of DIPGs, surgery is not an option making it the worst cancer diagnosis in this population with an abysmal 5-year survival of less than 1%. Hitherto, focal EBRT is the sole treatment option with a very limited clinical benefit, rather representing a palliative treatment for this devastating disease prolonging survival only from weeks to months [298,299]. Other therapeutic strategies such as CT, small-molecule inhibitors, and immunotherapies alone or combined with EBRT have not been successful. However, DIPGs present various targetable genetic alterations in H3, TP53, ACVR1, PIK3CA, FGFR1, or PDGFR, and efficacies of multiple-agent treatment approaches are currently tested in clinical trials [300].

##### Late Sequela and Second Primary Malignancies after Treatment of Pediatric Brain Cancer

The risk for sequelae after cranial EBRT for brain cancers and other tumor entities correlates with the size of the treatment volume, the cumulative radiation doses exceeding 25 Gy, and is inversely correlated with age [320]. Significant impairments in cognitive, neurological, endocrine, social, and emotional domains can occur, depending on the location and type of the treated brain tumor. Furthermore, survivors of a pediatric primary CNS tumor are at an about 7-fold increased risk to develop SPMs with higher incidences for patients treated later than 1985, most likely related to an intensification of therapy thereafter [321,322]. Strikingly, the risk for SPMs in survivors of pediatric CNS tumors continues to rise with prolonged follow-up and implies an even lifelong risk for these patients. For children treated with cranial EBRT for pediatric primary CNS tumors or leukemia at a median age of 8.1 years (range: 0.2–19.0), Galloway et al. [323] reported incrementing incidences of 3%, 8%, and 24% at 10, 20, and 30 years of follow-up, respectively. These data confirm previous observations during long-term follow-up in a Canadian cohort with cumulative SPM incidences of 3%, 11%, and 18% at 10, 20, and 30 years after the first diagnosis, respectively [324]. In the study of Galloway et al. [323], the average latency for SPMs was about 16 years and the vast majority of 40% of SPMs were diagnosed as meningiomas more than 20 years after the primary diagnosis. Early SPMs were more likely to be gliomas with high mortality rates. However, the overall survival and event-free 5 year survival for patients with EBRT-related meningiomas was 89%, indicating only limited mortality from SPMs in this study cohort [325]. Broniscer et al. [326] reported on the occurrence of SPMs with a cumulative incidence of 4% at 15 years follow-up in 1046 patients treated for pediatric and adolescent CNS tumors between 1984 and 2000. In this study, the authors did not find a correlation between SPM development and the applied treatment protocols. Rather, an association with genetic risk factors such as TP53 mutations, NF2, and Gorlin or Gardner syndrome has been described in line with the established causal relationship between the occurrence of multiple malignancies and various genetic syndromes in children with primary brain tumors [327]. Paugh et al. [315] investigated 10 HGGs arising as an SPM in children who received cranial EBRT for a primary tumor with nucleotide polymorphism microarray analysis. The molecular profiles of the EBRT-related SPMs were similar to other pediatric HGGs. Only an increased occurrence of chromosome 1q gain and PDGFRA amplification found in second primary HGGs were discussed as IR-induced initiating mutations, which are also known to promote sporadic pediatric HGGs. Based on the SEER incidence data from 1997–2007, Cai et al. [322] described an approximately twofold increase of excess risk of SPMs in pediatric patients with primary brain tumors who received EBRT compared to non-irradiated ones during the first 20 years of follow-up (19.43 versus 10.59, respectively). Surprisingly, after 20 years this trend was reversed and no significant difference in the risk of SPMs was observed between irradiated and non-irradiated patients. In a more recent study, Chojnacka et al. [328] investigated the development of SPMs in pediatric CNS patients who received EBRT at an average age of 4.6 years. Among 1404 children, only 9 received EBRT and developed an SPM as five meningiomas and four gliomas with a mean latency of 11.7 years. Seven of the SPMs developed in the treatment volume receiving radiation doses between 25–40 Gy and only two LGGs appeared in the low-dose region receiving less than 25 Gy. Therefore, the low-dose region does not seem to be most relevant for the development of second primary CNS tumors after cranial EBRT supporting the application of more conformal IMRT-based techniques. Although the successful therapy of pediatric primary CNS tumors is the major goal, there is an urgent need to lower the dose and volume of EBRT in the normal tissue and to organs at risk by highly dose-conformal IMRT, SBRT, and proton or hadron therapy in still physically and cognitively developing patients [320,329,330,331]. For pediatric CNS tumor patients, a large benefit is expected from the application of proton therapy allowing for a meaningful reduction of the dose burden to the healthy tissue with the option of simultaneous focal dose escalation in the tumor volume for highly resistant entities [35,320].

Hitherto, no long-term data after proton EBRT for pediatric CNS are available and the first results show no clear reduction of side effects or improved tumor control but clinical outcomes are comparable to photon EBRT and a long-term benefit is presumed [35]. Incrementing therapeutic response rates while reducing the side effects of conventional therapies in pediatric CNS tumor patients through the use of targeted and immunologic therapies is currently under investigation [332]. However, most pediatric brain tumors are considered ‘immune cold’, in particular, and unfortunately highly aggressive and lethal subtypes such as DIPG and MB, and the development of resistance to targeted therapies and cancer immune escape dampen the success of such therapeutic approaches.

#### 3.2.2. Sarcomas

STS occurring as RMS or nonrhabdomyosarcoma (NRMS) and malignant bone tumors represented by OS and ES account for about 14% of all childhood malignancies [10]. STSs make up 7% of total cancers in children and adolescents less than 20 years of age (4% RMS and 3% other STS) [10]. 40–50% of all STS are RMS, a tumor of the striated muscle of the embryonal type with favorable prognosis or the more clinically aggressive alveolar type as the most common and biologically distinct variants. NRMSs are a very heterogeneous group of rare mesenchymal tumors encompassing more than 50 different subtypes with distinct genetic profiles and phenotypes including tumors of the connective tissue (e.g., desmoid-type fibromatosis), peripheral nervous system (e.g., malignant peripheral nerve sheath tumor), smooth muscle (e.g., leiomyosarcoma), or vascular tissue-blood and lymphatic vessels (e.g., angiosarcoma) and are more frequent in adolescents and adults [333].

The development of a sporadic early-onset primary STS has been closely related to genetic predisposition and cancer risk syndromes. The most prominent risk factors are LFS, breast cancer, leukemia, and adrenal gland cancer syndrome [334]. Despite the relatively low incidence of primary pediatric STS, long-term survivors of primary STS account for a disproportionately high fraction of 20% of all patients developing an SPM, appearing predominantly as sarcomas, bone tumors, breast or thyroid cancer, and skin cancer without melanoma [177]. Second primary sarcomas have a very high clinical significance in childhood cancer survivors showing a nine-fold higher risk for their development compared to the general population [177]. Second primary sarcomas represent the second most common SPM after leukemia in pediatric tumor survivors and general risk factors are a primary diagnosis of HL, CNS or kidney tumor, sarcoma, EBRT, higher doses of anthracyclines or alkylating agents, and the history of another SPM [61]. This high incidence of sarcomas as SPMs that reflects the LFS spectrum is closely linked to the aforementioned genetic predisposition in this malignancy and its interaction with the genotoxic impact of CT and EBRT. The development of second primary sarcomas is a well-recognized late complication of EBRT correlating with the radiation dose [335] and occurring most frequently as undifferentiated pleomorphic or spindle-cell sarcoma, OS, and angiosarcoma [336,337]. The development of approximately 3–6% of total sarcomas has been associated with a previous EBRT [338,339] with a median latency of 12 years (range: 4.1–15.5) [337,340]. An overview of pediatric primary STS, risk factors for their development, current treatments, non-cancerous late effects as well as the most common SPMs, and risk factors for their formation is provided in Table 3.

##### Rhabdomyosarcoma

Strikingly, first-degree relatives of pediatric RMS patients have a 1.4-fold elevated incidence of cancer, and patients developing a tumor below the age of 30 years are 2.4 times more likely to have a first-degree relative with RMS [341,342]. Further associations were found between RMS and germline DICER1 mutations, Beckwith–Weidemann syndrome, Costello syndrome, and Noonan syndrome [343,344,345]. Despite a generally low mutation rate, RMS tumor specimens are characterized by specific genomic alterations. Most dominant are the chromosomal translocations t(2;13)(q35;q14) or t(1;13)(q36;q14) in alveolar RMS causing the expression of chimeric proteins PAX3-FOXO1 in 60% or PAX7-FOXO1 in 20% of patients, respectively [346]. PAX3-FOXO1 fusions are a crucial prognostic indicator in this disease but coexisting genetic lesions are necessary to cause RMS [380]. The genomic landscape of RMS further includes genetic aberrations in FGFR4, IGF1R, PDGFRA, ERBB2/4, MET, MDM2, CDK4, and PIK3CA and BCOR providing targets for molecular therapies for this tumor type [345]. The 5-year overall survival of patients with pediatric RMS now exceeds 70% due to continuous improvements of multimodal therapies [349,381,382] whereas the overall survival rates for the metastatic or recurrent disease remain below 30% with no significant improvements in the last 30 years [383,384].

Frontline therapy for all RMS groups is a multimodal approach with CT, surgery, and/or EBRT. Common CT protocols consist of VAC (vincristine, actinomycin D, and cyclophosphamide), or IVA (ifosfamide, vincristine, and actinomycin D) [349,350]. For patients with localized, low-risk disease and good prognosis, a current goal is the reduction of cyclophosphamide as a well-known inductor of acute and late effects including severe myelosuppression, infectious complications, and infertility [354,355]. EBRT is essential for the treatment of almost all RMS patients but increases the risk and incidence of long-term consequences [347]. For the SEER-9 cohort (1973–2014), Archer et al. [353] reported a 5.6- and 15.8-fold increase of SPMs for pediatric survivors of pleomorphic and embryonal RMS, respectively. The treatment of pleomorphic RMS by EBRT increased the risk tremendously up to 300-fold but EBRT did not seem to increase the risk in their overall population. EBRT for head and neck RMS may affect visual, endocrine, cardiopulmonary, neurosensory, and neuromotor sequelae more than 5 years after the initial diagnosis and is a significant contributor to neuroendocrine, dental, thyroid, and cognitive toxicity [351,352]. To reduce side- and late-effects of conventional 3D-CRT, IMRT and proton therapy are currently applied as highly conformal EBRT techniques to treat RMS patients but further clinical studies and extended follow-up are needed to evaluate acute toxicities and long-term outcomes of such treatment modalities [348].

##### Nonrhabdomyosarcoma

NRMSs are categorized as tumors with gross genomic instability or with specific chromosomal aberrations and translocations that have been related to their pathogenesis [333]. e.g., synovial sarcoma is associated with t(x;18)(p11;q11) (SYT-SSX), alveolar soft parts sarcoma is associated with t(X;17)(p11;q25) (ASPL-TFE3) or myxoid liposarcoma with t(12;16)(q13;p11) (TLS-CHOP) [356]. NRMSs are found most commonly in adults and predisposing factors include previous exposure to IR or genetic syndromes, such as NF, LFS and Maffucci syndrome [333].

Despite a large diversity, the group of these tumors that occurs most often in the extremities of the lower limb is commonly treated by similar regimes of surgery, CT, and EBRT based on location, size, stage, and class. Resistance to CT is observed frequently and adjuvant CT for pediatric NRMS patients usually compromises doxorubicin, vincristine, cyclophosphamide, and dactinomycin [357]. Neoadjuvant or adjuvant EBRT is applied as conventional 3D-CRT or IMRT to achieve local tumor control in patients with large and high-risk tumors and has been associated with adverse side effects like subcutaneous fibrosis, lymphedema, and joint stiffness [358]. Long-term consequences include cardiac, skeletal, renal, and fertility sequelae and SPMs [385]. Attempts are being made to reduce the side effects of EBRT for NRMS patients through highly conformal EBRT techniques such as IG-IMRT or proton EBRT [386].

Clinical trials investigate the efficacies of therapies targeting molecular structures like IGF-1R (cixutumumab), PDGFR (pazopanib, sorafenib), VEGF (pazopanib, bevacizumab, sorafenib), ALK (crizotinib), MET (crizotinib), and mTOR (temsirolimus) to lower treatment-related adverse effects and to improve outcomes in patients with sarcomas including NRMSs [348,359]. Also, immuno-oncology may be a promising approach for the treatment due to a correlation of PD-L1 expression in various sarcomas and poor overall survival [360] and monoclonal antibodies targeting PD-L1 (nivolumab) have already been used in clinical trials to treat pediatric sarcoma patients [361]. Further immune-based strategies rely on the anti-tumor activation of cytotoxic T-lymphocytes by antigen-presenting dendritic cells primed with tumor antigens or the adaptive T-cell therapy (ACT) with CAR-T cells targeting NY-ESO-1 or HER2 [348]. Promising response rates to these therapies have been observed in patients with pediatric primary sarcomas, but small population sizes and large heterogeneity of tumor entities in these studies do not yet allow a conclusion on their effectiveness [387,388].

##### Osteosarcoma

OSs represent 30–80% of primary skeletal sarcomas and primarily affect children, adolescents, and young adults aged 10–30 years [389]. Predisposition for OS is observed in patients with hereditary RB, Rothmund–Thomson syndrome, LFS, and WRN but the majority of OSs are observed in patients with no known germline alterations [362]. Some other somatic alterations in oncogenes associated with amplifications in OS include CDC5L, MAPK7, MET, PIM1, PMP22, PRIM1, RUNX2, and VEGFA [363].

Currently, the event-free survival of OS patients is about 60–70% after neoadjuvant CT comprising cisplatin, doxorubicin followed by high-dose methotrexate, and surgical removal of the tumor often followed by adjuvant CT. EBRT plays no or only a minor role due to the high radioresistance of OS and is only applied on rare occasions when surgical margins are limited by tumor localization with an additional positive impact on prognosis, but high doses up to 80 Gy may be required to achieve some additional benefit [390]. Here, recent studies using highly localized combined ion-beam EBRT for inoperable OS showed promising results and may represent a treatment option with a favorable toxicity profile [364].

Survivors of OS are at a general 2.7-fold increased risk of developing SPMs compared to the general population, unfortunately with an increasing trend in the most recent era as reported by Lee et al. [366] based on the SEER database from 1973–2010. The SIR for SPMs of survivors of OS increased from 1.6 for patients diagnosed between 1973–1985 to 4.7 for patients diagnosed between 1986–2010 with a 34-fold increased risk of leukemia compared with the general population. This is attributed to the use of high-dose CT, which provides a survival benefit for OS patients but increases the risk for SPMs, usually presenting as tumors of the hematopoietic system, soft tissue, thyroid, respiratory system, and breast. The cumulative incidences of SPMs in survivors of a primary OS are 2.1% at 10 years, 4.0% at 20 years, and 7.4% at 30 years after the initial diagnosis with an average latency of 6 years (range: 0.6–36).

OSs are among the most abundant SPMs in childhood cancer survivors [391]. The highest rates and about 25% of second primary OSs are observed in patients treated by EBRT for RB [392] but also occur after a primary Ewing’s tumor, RMS, NB, Wilms’ tumor, and Hodgkin’s disease [393]. Besides increased risk for a second primary OS after EBRT only in the exposed bones [394], there is also elevated risk after CT with alkylating drugs and anthracyclines with an overall latency ranging between 6–20 years and a poor prognosis [393,395].

##### Ewing Sarcoma

ES is an aggressive tumor in adolescents and young adults with the highest incidence between 10–15 years of age and with 30% occurring in children under the age of 10 years. ES accounts for about 2% of total childhood cancers, is the second most common primary bone malignancy in adolescents and young adults after OS, and constitutes 10–15% of all bone sarcomas [369,396]. Hitherto, no association could be established between ES and environmental risk factors, drug exposure, IR, or cancer history in the family. Familial ESs are characterized by the chromosomal translocation t(11;22)(q24;q12) causing EWS-FLI-1 formation or translocations inducing an EWS-ERG fusion in 85% or 15% of tumors, respectively [367].

The application of EBRT, surgery and multiagent CT achieves 5-year survival rates of 70% for localized ESs, but recurrences and metastatic disease with very poor prognosis occur frequently in about 25% of patients with survival rates of less than 30% [369].

The treatment of ES is based on definitive local therapy by EBRT and radical surgery followed by VACD (vincristine, cyclophosphamide, Actinomycin D, and doxorubicin) CT plus ifosfamide and etoposide leading to improved event-free survival for patients with localized, but not metastatic disease. Unfortunately, also high-dose CT supported by autologous bone marrow transplantation does not improve survival rates and can cause treatment-related AML and MDS observed at an incidence of about 8% [377,378].

According to the study by Sultan et al. [379] for the SEER-9 database (1973–2014), SPMs were observed in 3% of total ES patients with a 4–9-fold higher risk compared to the general population. Treatment with EBRT increased the risk of SPMs, and children and adolescents had about twice the risk compared with adults. Based on a study by Marina et al. [397] on 404 5-year survivors of ES within the childhood cancer survivors study (CCSS) treated between 1970 and 1986, SPMs occurred with a cumulative incidence of 14.3% at 35 years primarily as OS (SIR 377.1), AML (SIR 28.9), breast cancer (SIR 14.9), and thyroid cancer (SIR 13.1). Comparable results were obtained by Lin et al. [398] for an updated analysis of the SEER-9 (1973–2014) database [379,399] but with higher incidences for hematologic SPMs since a 5-year lag time before the development of an SPM as applied for the CCSS cohort was not allowed. Together, survivors of ES have a significantly increased risk for solid and hematologic SPMs. Despite a high radiosensitivity of ESs, EBRT is used less frequently to reduce the risk of SPMs or adverse effects on bone growth and more advanced surgical techniques are recommended for local treatment [400,401]. However, neoadjuvant EBRT is highly beneficial in patients with limited surgical options and positive or close tumor margins but local failures still occur in approximately 20% of adults and adolescents mainly in the EBRT field, and more aggressive multidisciplinary approaches with dose-escalation are recommended for adults [402,403]. Proton therapy has been rarely applied in pediatric ES patients to achieve sufficient local intensity where complete surgery is complicated and to minimize the risk of side effects and SPMs. Encouraging preliminary results were obtained by Rombi et al. [404] for the local treatment of 30 pediatric ESs with proton therapy. After a follow-up of 3 years, their data show an event-free survival, local control, and overall survival of 60%, 86%, and 89%, respectively. The cumulative incidence of SPMs was 7% at 2 years and 15% at 3 years after treatment and data on long-term follow-up are highly warranted. No solid SPMs were observed but only cases of second primary AML (*n* = 4) and MDS (*n* = 1), most probably associated with the use of high cumulative doses of etoposide. In general, the use of VACD plus etoposide and ifosfamide increase the risk for second primary MDS and AML in ES patients about 16-fold with a cumulative incidence of 11% at 5 years compared to VACD only with a cumulative incidence of 0.4% and 0.9% at 5 years, respectively [405]. For metastatic ES, the use of multisite SBRT may represent a successful strategy to improve event-free survival [368].

Several molecular candidates are postulated in ES for therapies targeting the EWSR1/FLI1 fusion protein or the downstream transcriptional product EZH2 (tazemetostat), BET proteins (BRD2, BRD3, BRD4), LSD1, NKX2.2 via an HDAC inhibitor (vorinostat), CDK4/6, EWSR1/FLI1 (trabectedin, lurbinectedin), the IGF1/IGF1R-axis (cituximab, figitumumab) combined with a mTor1-inhibitor (temsirolimus), ERK or HSP90 inhibitors, the DNA damage response with PARP inhibitors (olaparib, talazoparib, niraparib) with or without CT, VEGFR (cediranib, regorafenib) or the TRKs c-KIT and PDGFR (imatinib, regorafenib) [370].

Immunological strategies are also explored to improve the treatment of ES [406]. However, ESs are generally considered as immunologically inert tumors with a low mutational burden and lack of high-affinity neoepitopes, low PD-L1 expression, lack of potentially tumor-reactive T cells in the tumor microenvironment, and HLA loss [371,372,373]. Accordingly, clinical strategies using the ICI pembrolizumab directed against PD-1 in adults with ES did not show significant clinical activity [374] but further studies are investigating the efficacy of ICIs in bone sarcoma therapy [375]. Alternative immunological signaling pathways are being considered as clinical targets in ES compromising the CXCR4-CXCL12 axis targeted by the CXCR4-antagonist AMD3100, intracellular antigens including WT1, XAGE-1, and targets of the EWS–FLI1 chimeric transcription factor (e.g., FATE 1), cancer vaccines based on PAX3- and EWS–FLI1-epitopes specifically expressed in ES tumor cells, vigil immunotherapy, oncolytic viruses, T cell receptor therapy with anti-CD3/4-1BBL cells or CD8+ T cells targeting antigens like PAPPA, EZH2 or CHM1, CAR-T cells targeting LINGO, ROR1 or IGF-1R, the latter may also be blocked by monoclonal antibodies and others which are currently tested or considered for future clinical trials [406].

ES itself has been rarely diagnosed as an SPM after various unrelated primary pediatric malignancies including lymphoma, leukemia, RB, or Wilms tumor [407,408,409]. Spunt et al. [409] observed second primary ESs in 1.3% of 11,183 children and adolescents treated for different primary malignancies and Appelbaum et al. [407] accounted second primary ESs for 2.1% of all ESs in the SEER database (1973–2008) with a median latency of 64 months (range: 1–282). In contrast to other bone tumors, EBRT, any specific CT, or the type of primary cancer do not seem to be risk factors for the development of second primary ES. Approximately 2/3 of ES presenting as SPMs are associated with EWSR1 gene rearrangements, suggesting a sporadic rather than therapy-induced occurrence [410,411].

#### 3.2.3. Other Tumor Entities

Finally, we give an overview of other relevant pediatric tumor entities including neuroblastoma, Wilms tumors, and retinoblastoma. A survey on the risk factors for their development, current treatments, non-cancerous late effects as well as the most common SPMs, and risk factors for their formation is provided in Table 4.

##### Neuroblastoma

NB is a developmental neoplasm of the autonomic nervous system primarily affecting young children [412]. NBs show a broad heterogeneity in clinical response ranging from spontaneous regression to fatal outcomes despite intense multimodal therapy [413]. Based on patient age, post-surgical stage, MYCN amplification, histology, and DNA ploidy, NB patients are classified into low-, intermediate- or high-risk and tumor stage 4S [427]. Genetic factors fostering NB onset and progression include amplification of MYCN, deletions of TP53, mutations or amplifications of ALK, rearrangements of TEEBRT, and deletions or mutations of ATRX [412].

Low- and intermediate-risk NBs show very slow growth rates, or spontaneous regression and watch and wait approaches proved that about 57% of patients with localized NBs can be spared from surgery or CT to avoid significant adverse effects and morbidity without hampering the overall high survival rate of 90–95% [427]. However, for disseminated and treatment-resistant high-risk NB in non-infants, the survival rate is below 40% despite intensive CT, multiagent myeloablative regimens, surgery, EBRT, and immunotherapy [413]. Intense CT comprising cyclophosphamide, doxorubicin, vincristine, cisplatin, etoposide, and prolonged exposure to oral etoposide caused high therapy-related death rates as well as SPMs occurring as leukemia [336,337]. Besides, EBRT and myeloablative therapy with stem cell rescue further increase the risk of treatment-related second primary hematologic malignancies. The overall cumulative incidence of SPMs at ten years for high-risk patients is 1.8% compared to 0.38% for low-risk patients [415]. Besides leukemia, SPMs occur as sarcomas, carcinomas, or brain tumors. Genetic alterations in the DNA repair factors XRCC3 and MSH2 have been identified as possible predisposing genetic determinants [415].

Surprisingly, tumor stage 4S as a widely disseminated disease in infants shows favorable outcomes with survival rates higher than 90% due to spontaneous regression of the tumor as a delayed step of cellular differentiation and apoptosis of tumor nodules [428].

##### Wilms Tumor

Wilms tumor (nephroblastoma) is the most common malignant renal tumor in childhood affecting one in 10,000 children per year. In more than 80% of cases, Wilms tumors are diagnosed in children at a median age of 3.5 years [429]. The causes for Wilms tumors are not precisely known, but gene alterations of WT1, CTNNB1, and WTX have been found in about 30%. Other genes associated with Wilms tumors include TP53 and MYNC [416]. The development of Wilms tumors is also associated with several syndromes like WAGR, Drash, Beckwith-Wiedemann, Sotos, Perlman, Edward’s, Frasier, BLM, LFS, and Simpson-Golabi-Behmel. Long-term survival of Wilms tumor patients has improved dramatically, with a 5–7-year survival of 30% just a few decades ago to over 90% today, with simultaneous reduction of genotoxic therapies for the majority of patients. The common treatment approach is a combination of surgery and CT comprising vincristine and dactinomycin in children with localized tumors plus doxorubicin in metastatic disease plus EBRT for high-risk patients [417,418].

The most common long-term complications in survivors of Wilms tumor are cardiotoxicity (4.4%), musculoskeletal problems (3%), and SPMs (1%) [417,418,421]. The administration of anthracyclines like doxorubicin is the highest risk factor for congestive heart failure in these patients. EBRT for Wilms tumors can compromise growth and development, musculoskeletal functions and increases the risk for radiogenic lung fibrosis [430]. The risk for SPMs in long-term survivors of Wilms tumors has been estimated to be about 6.7% at 40 years from diagnosis [421] and the most frequent SPMs include bone and soft-tissue sarcomas, breast cancer, lymphoma, leukemia, and melanoma [417].

Genetic aberrations in Wilms tumors discussed as potential targets for molecular interventions include WT1, CTNNB1, WTX, TP53, FBXW7, MYCN, SIX 1/2, DICER1, DROSHA, DGCR8, and IGF2, but preclinical data has become available only recently [431]. Promising target structures are antiangiogenic compounds, inhibitors to aurora-A-kinase, the mTOR pathway, c-Met, JAK2, cell cycle, telomerase, HER2, ATR, and in particular the WNT signaling pathway which is mutated in 35% of Wilms tumors as previously mentioned [432]. Recent clinical trials showed durable responses for the combination of TRK inhibitors and monoclonal antibodies directed against PD-1 (nivolumab, pembrolizumab) for advanced treatment-refractory renal cell carcinoma which might be implemented into therapeutic strategies for Wilms tumors [419,420].

Renal cancer is generally very rare in individuals below 40 years of age but is seen as SPMs in childhood cancer survivors with statistically significant excess (SIR 8.0) compared with the general population as reported by Wilson et al. [433] for the CCSS. The most obvious risk factor is a previous therapy of a primary NB with renal-directed EBRT of 5 Gy or higher and platinum-based CT. The administration of alkylating agents has been associated with second primary renal cancers characterized by Xp11.2 translocations and TFE3 gene transfusions [434,435].

##### Retinoblastoma

RB is the most common intraocular malignancy in childhood. In 95% of cases, RB is caused by biallelic mutation of the RB1 tumor suppressor that initiates additional genetic and epigenetic changes, paradigmatically representing genetic cancer caused by inactivation of tumor suppressor genes [422]. Heritable RB with a germline mutation in one allele of the RB1 gene followed by an acquired mutation in the second allele accounts for about 45% of all cases, with 80% being bilateral. RB is diagnosed in about 8000 children annually worldwide but survival rates are strongly influenced by the socio-economic status and therapeutic options of a country. They are higher than 95% in high-income countries but less than 30% globally [436].

Primary treatments for intraocular disease include enucleation, intravenous CT (melphalan with or without topotecan and/or carboplatin) with focal therapy (laser therapy, cryotherapy), intra-arterial CT with focal therapy, and focal therapy alone when tumors are small at diagnosis [423]. The choice of treatment is based on the likelihood of tumor control, eye salvage, ultimate vision, and the status of the other eye weighed against acute and chronic consequences of treatment. EBRT has been extensively used since the 1960s but is no longer recommended due to the therapeutic success of CT and because of severe side effects such as SPMs and other EBRT-related complications [424,425]. RB patients with germline RB1 mutation who received EBRT have a high risk of 50% to develop SPMs with advanced age including leiomyosarcoma, OS, melanoma, lung, and bladder cancer [426]. Currently, several molecular targets such as SKP2 (e.g., via the NEDD8-inhibitor pevonedistat), MDM2, histone deacetylases, or the TRK SYK are being discussed for the treatment of CT-resistant RB to improve cure rates while reducing treatment-related side effects [423].

## 4. Conclusions

Childhood cancer itself is a devastating disease, and intensive multimodal therapies put long-term survivors at high risk for severe and even fatal late health consequences including SPMs. With a continuously increasing number of long-lived cancer survivors, this issue is becoming more and more clinically relevant, in particular for childhood cancer patients. Besides iatrogenic non-cancerous late sequelae, SPMs represent the heaviest burden for the patient. Therefore, it is of great importance to assess and stratify risks of SPMs, taking into account the impact of CT, RT, and a possible, as yet unknown influence of targeted and immunological therapies and their multimodal combinations, as well as of known intrinsic factors to allow for intense follow-up with structured survivorship care. For the reduction of late sequelae in cancer survivors, the use of highly conformal and locally intensified EBRT techniques as well as targeted therapies and immunological treatments with non-genotoxic mechanisms of action are currently the most promising approaches. However, to date, these mostly personalized treatments are rarely applied for the scarce cases of different pediatric tumor entities with a limited time since application and a clinical benefit with concomitant reduction of late adverse health effects is not yet predictable. Besides, the success of targeted therapies and immunologic treatments is frequently dampened by low and heterogeneous response rates, the development of therapy resistance, and cancer immune escape. Consequently, conventional EBRT and CT continue to have their dominant role in the treatment of pediatric tumors. Thus, there is an urgent need to decipher the etiology of SPMs and to establish predictive clinical biomarkers for individual susceptibility to therapy-related SPMs to adapt oncologic treatments and intensify follow-up with intervention strategies and multidisciplinary care. Next-generation sequencing approaches and affected pathway analysis may unravel molecular genetic markers based on which, e.g., functional bioassays can be performed on minimally invasively obtained normal tissue samples for risk assessments in any given individual [437,438,439]. This provides an opportunity to identify high-risk patients who will benefit from close surveillance and ultimately to curtail therapy-related SPMs through a mechanistic understanding of their development by targeted interventions.

## Figures and Tables

**Table 1:** Overview of the major primary hematologic tumors of childhood, risk factors for their development, current treatments, non-cancerous late effects, most common second primary malignancies, and risk factors for their evolvement.

Primary Hematological Tumor			Late Effects		
Entity	Predisposition and Risk Factors	Treatment	Non-Carcinogenic	Second Primary Malignancies	Risk Factors
Acute lymphoblastic leukemia	DS, FA, AT and Klinefelter syndrome [120,121] translocations causing TEL-AML1, MLL and MLLAF4 gene fusions [122,123]	CT (prednisone, cyclophosphamide, cytarabine, dexamethasone, etoposide, ifosfamide, methotrexate, doxorubicin, mercaptopurine, vincristine) intrathecal therapy [124,125,126] cranial or craniospinal EBRT [127] targeting CD19 and T-cell receptor-CD3 complex (blinatumomab), cytotoxic anti-CD22-calicheamicin conjugate (inotuzumab ozogamicin), anti-CD22 or -CD19 CAR-T cell-based therapies (e.g., with tisagenlecleucel), TRK inhibitors (dasatinib, ruxolitinib, crizotinib) [128,129,130]	growth, neurocognition [131,132,133,134,135]	meningioma, medulloblastoma, primary leukemia, lymphoma, thyroid, breast and bone cancer, soft tissue sarcoma, squamous cell carcinoma of the skin [12,133,136]	cranial EBRT [134] CT with alkylators (cyclophosphamide), topoisomerase II inhibitors (epipodophyllotoxins), high-dose therapy with methotrexate and mercaptopurine [135]
Acute myeloid leukemia	DS, NF, LFS, FA, and Klinefelter’s syndrome [137] viruses, IR, chemicals, or previous CT [137] loss or deletion of chromosome 5, 7, Y, and 9, t(8;21)(q22;q22), t(15;17)(q22;q11), or trisomy 8 and 21 [138] genetic alterations in TP53, RUNX1, IKZF1, and ETV6 [139]	CT (anthracyclines and cytarabine) [140,141] reduced EBRT [142] anti-CD33-calicheamicin conjugate (gemtuzumab ozogamicin), TRK inhibitors (sorafenib, midostaurin, gilteritinib) targeting FLT3-ITD TRK mutations [143,144,145,146]	endocrine abnormalities, cataracts, cardiac abnormalities, growth-hormone deficiency, hypothyroidism	Very rare due to death by any other cause mucoepidermoid carcinomas, supratentorial primitive neuroectodermal, ALL, NHL [147]	high dose CT cranial EBRT [147]
Non-Hodgkin lymphoma	infection and immune dysregulation [148] probably alterations in TNF and IL10, toll-like receptor or RAG1, LIG4, ERCC5, WRN, MGMT, and XRCC1 [148]	NHL-BFM and cranial EBRT if CNS involved plus CNS prophylaxis with intrathecal CT and methotextrat [149,150] rituximab targeting CD20 on B cells, antibody-drug conjugates (inotuzumab-ozogamicin, polatuzumab-vedotin, pinatuzumab-vedotin), CAR T-cell therapy (tisagenlecleucel), BTK inhibition (ibrutinib), T-cell-engaging antibody constructs (blinatumomab, mosunetuzumab) [151,152,153,154,155,156]	cardiac disease, pneumonia [157]	carcinomas, acute myeloid leukemia, lymphoid malignancies [157,158]	high doses of alkylators, topoisomerase II inhibitors, and anthracyclines [158] sex (female), lymphoblastic lymphoma, CNS involvement, AT, NBS, and constitutional mismatch repair deficiency [158]
Hodgkin lymphoma	Epstein-Barr-Virus, genetic factors, immune-related disorders, other infections, environmental exposures, familial predisposition [159,160]	involved-field/involved node EBRT CT (OP/EPA [vincristine, prednisone, procarbazine/etoposide, and doxorubicin] or OPPA/COPP [cyclophosphamide, vincristine, prednisone, and procarbazine]) [161] CD30-directed brentuximab vedotin, PD-1 (pembrolizumab, nivolumab) JAK2 inhibitors (itacitinib, ruxolitinib), CAR-T cells, histone deacetylases inhibitors(panobinostat), immunomodulatory drugs (lenalidomide), BTK inhibitors (ibrutinib), mTOR inhibitors (everolimus), CD25-directed antibody-drug conjugates (camidanlumab tesirine) [162,163,164]	pulmonary, dysfunction, endocrinopathies, thyroid dysfunction, infertility, neck muscle atrophy, persistent fatigue [165]	thyroid carcinoma, breast cancer, lung cancer, sarcoma, colorectal carcinoma, melanoma, cervix carcinoma [53,55,166,167,168,169]	EBRT, particularly chest exposures for breast cancer in females and lung cancer in males and alkylating drugs [55,167,170,171]

*Abbreviations*: SPM, second primary malignancy; DS, Down syndrome; NF, neurofibromatosis; LFS, Li-Fraumeni syndrome; FA, Fanconi anemia; CT, chemotherapy; EBRT, external beam radiotherapy; ALL, acute lymphoblastic leukemia; NHL, non-Hodgkin lymphoma; CNS, central nervous system; AT, ataxia telangiectasia; NBS, Nijmegen breakage syndrome.

**Table 2:** Overview of the major primary childhood brain tumors, risk factors for their development, current treatments, non-cancerous late effects, most common second primary malignancies, and risk factors for their evolvement.

Primary Brain Tumor			Late Effects		
Entity	Predisposition and Risk Factors	Treatment	Non-Carcinogenic	SPM	Risk Factors
Medulloblastoma	Turcot syndrome, Gorlin syndrome, Rubinstein Taybi syndrome, LFS and FA alterations in WNT, SHH, MYC, PVT1, SMARCA4, OTX2, and abnormalities of chromosome 17 [248,249]	surgery and adjuvant craniospinal EBRT [250] CT with vincristine and cisplatin plus either lomustine or cyclophosphamide [251] SHH pathway-inhibitors (saridegib, erismodegib, or vismodegib) [249], targeting CDK4/6, c-Met, Wee1, PI3K/mTOR, EZH2, CHK1/2, or the BET bromodomain pathways [248], PD-1 inhibitors (pembrolizumab, nivolumab), monoclonal antibodies against CD40 (APX005M), PEP-CMV (cytomegalovirus) based vaccine trials for oncolytic viral therapy [248]	hypothyroidism, adrenocorticotropic, hormone deficiency, altered metabolism [252,253]	glioma, meningioma, thyroid carcinoma [254,255,256]	CT and craniospinal EBRT
Ependymoma	infection with SV40 virus, NF2, Turcot syndrome B, LFS [257,258,259]	maximal surgical resection and adjuvant EBRT [260,261,262] proton beam EBRT [260,263] CT with platinum derivatives, etoposide, cyclophosphamide, vincristine, and methotrexate, (so far not superior to adjuvant EBRT) [264], inhibition of ERBB1 and ERBB2 (lapatinib), interference with the NFκB pathway, inhibition of YAP1 [265]	neurocognitive impairment, neurologic deficits, neuroendocrine deficiency [266]	known for adults: pancreatic cancer, prostate cancer, Hodgkin lymphoma, intracranial meningioma, pulmonary adenocarcinoma, gastric cancer, astrocytoma [267]	intrinsic risk factors [267] hypermethylated phenotype causing silencing of CDKN2A, CDKN2B, HIC1, RASSF1A, CASP8, MGM, and TP73 [268,269]
Low-grade gliomas	NF1, tuberous sclerosis, complex germline mutations [270]	surgery with no adjuvant therapy after complete tumor resection [271] CT with carboplatin and vincristine, TPCV (thioguanine, procarbazine, lomustine, and vincristine), weekly vinblastine monotherapy, BRAF-inhibitors (vemurafenib, dabrafenib, trametinib), MEK-inhibitors (selumetinib), mTOR inhibitors (everolimus), VEGF-inhibitors (bevacizumab) [272,273,274,275]	neurocognitive function, neuroendocrine deficiency, vasculopathy		EBRT [276]
High-grade gliomas	compromised TP53, CDKN2A, PI3K/TRK, and RB pathway [277,278,279,280,281]	radical surgery followed by focal EBRT with temozolomide [282] BRAF and MEK inhibitors, pan-TRK inhibitors, monoclonal anti-PD-1 antibody (nivolumab) in MMR deficient patients [283,284,285,286,287,288]	neurocognitive function, neuroendocrine function, vascular changes leading to increased stroke risk [289]	hematologic, meningioma, gliomas	EBRT [289,290,291] temozolomide and other alkylating drugs [292,293,294,295,296]
Diffuse intrinsic pontine gliomas	mutations in histone variants H3F3A or HIST1H3B [297]	focal EBRT [298,299] targeting alterations in H3, TP53, ACVR1, PIK3CA, FGFR1, or PDGFR [300]	almost universally fatal		

*Abbreviations*: SPM, second primary malignancy; NF, neurofibromatosis; LFS, Li-Fraumeni syndrome; FA, Fanconi anemia; CT, chemotherapy; EBRT, external beam radiotherapy.

**Table 3:** Overview of major primary childhood sarcomas, risk factors for their development, current treatments, non-cancerous late effects, most common second primary malignancies, and risk factors for their evolvement.

Primary Sarcoma			Late Effects		
Entity	Predisposition and Risk Factors	Treatment	Non-Carcinogenic	SPM	Risk Factors
Rhabdo-myosarcoma	first-degree relatives of pediatric RMS patients, LFS, germline DICER1 mutations, Beckwith Weidemann syndrome, Costello syndrome, Noonan syndrome, and genetic aberrations in FGFR4, IGF1R, PDGFRA, ERBB2/4, MET, MDM2, CDK4, PIK3CA and BCOR expression of PAX-FOXO chimeric proteins [341,342,343,344,345,346]	surgery and/or EBRT [347,348] CT by VAC (vincristine, actinomycin D, and cyclophosphamide) or IVA (ifosfamide, vincristine, and actinomycin D) [349,350]	visual, endocrine, cardiopulmonary, neurosensory, neuromotor, neuroendocrine, dental, thyroid, cognitive [351,352]	sarcomas, bone tumors, breast or thyroid cancer, or skin cancer without melanoma [177]	EBRT [353] cyclophosphamide [354,355]
Nonrhabdo-myosarcoma	NF, LFS and Maffucci syndrome translocations causing SYT-SSX, ASPL-TFE3, and TLS-CHOP fusion previous exposure to IR [333,356]	CT with doxorubicin, vincristine, cyclophosphamide, and dactinomycin [357] EBRT [358] targeting of IGF-1R (cixutumumab), PDGFR (pazopanib, sorafenib), VEGF (pazopanib, bevacizumab, sorafenib), ALK (crizotinib), MET (crizotinib), mTOR (temsirolimus), PD-L1 (nivolumab), or CAR-T cells targeting NY-ESO-1 or HER2 [348,359]	subcutaneous fibrosis, lymphedema, joint stiffness, cardiac, skeletal, renal, infertility [358,359,360,361]		EBRT [353] cyclophosphamide [354,355]
Osteosarcoma	RB, Rothmund-Thomson syndrome, LFS, and WRN [362] amplifications in CDC5L, MAPK7, MET, PIM1, PMP22, PRIM1, RUNX2, and VEGFA [363]	neoadjuvant CT comprising cisplatin, doxorubicin followed by high-dose methotrexate, and surgical removal of the tumor often followed by adjuvant CT conventional high dose EBRT rarely applied, ion-beam EBRT for inoperable osteosarcoma [364]	cardiac toxicity nephrotoxicity, neurotoxicity, hearing loss, infertility [365]	hematopoietic, soft tissue, thyroid respiratory system, breast [366]	EBRT and high dose CT [366]
Ewing Sarcoma	translocations causing EWS-FLI-1 or EWS-ERG gene fusions [367]	EBRT and radical surgery SBRT [368] multiagent CT with VACD (vincristine, cyclophosphamide, Actinomycin D, and doxorubicin) CT plus ifosfamide and etoposide [369] targeting EWSR1/FLI1 or EZH2 (tazemetostat), BET proteins (BRD2, BRD3, BRD4), LSD1, NKX2.2 (vorinostat), CDK4/6, EWSR1/FLI1 (trabectedin, lurbinectedin), the IGF1/IGF1R-axis (cituximab, figitumumab) combined with a mTor1-inhibitor (temsirolimus), ERK or HSP90, PARP (olaparib, talazoparib, niraparib), VEGFR (cediranib, egorafenib), or c-KIT and PDGFR (imatinib, regorafenib) [370] immunological signaling pathways compromising CXCR4-CXCL12 (AMD3100), intracellular antigens including WT1, XAGE-1, the EWS–FLI1 chimeric transcription factor, cancer vaccines based on PAX3- and EWS–FLI1, vigil immunotherapy, oncolytic viruses, T cell receptor therapy with anti-CD3/4-1BBL cells or CD8+ T cells targeting antigens like PAPPA, EZH2 or CHM1, CAR-T cells targeting LINGO, ROR1 or IGF-1R [371,372,373,374,375]	cardiac, pulmonary, bone growth, musculoskeletal, infertility [376]	acute myeloid leukemia, myelodysplastic syndromes, breast and thyroid cancer [377,378,379]	EBRT VACD plus etoposide and ifosfamide [377,378]

*Abbreviations*: SPM, second primary malignancy; NF, neurofibromatosis; LFS, Li-Fraumeni syndrome; FA, Fanconi anemia; RB, retinoblastoma; WRN, Werner syndrome; SBRT, stereotactic body radiotherapy; CT, chemotherapy; EBRT, external beam radiotherapy; IR, ionizing radiation.

**Table 4:** Primary childhood neuroblastoma, Wilms tumor, and retinoblastoma, risk factors for their development, current treatments, non-cancerous late effects, most common second primary malignancies, and risk factors for their development.

Primary tumor			Late Effects		
Entity	Predisposition and Risk Factors	Treatment	Non-Carcinogenic	SPM	Risk Factors
Neuroblastoma	alterations of MYCN, TP53, ALK, TEEBRT, and ATRX [412]	watch and wait for low-risk [412] CT comprising cyclophosphamide, doxorubicin, vincristine, cisplatin, etoposide, and prolonged exposure to oral etoposide, multiagent myeloablative regimens, surgery, EBRT, and immunotherapy for intermediate- and high-risk as well as few low-risk patients [413]	musculoskeletal, pulmonary, hearing loss, primary hypothyroidism, cardiovascular [414]	leukemia, sarcoma, carcinoma, or brain tumors [336,337,415]	intense CT and prolonged exposure to oral etoposide, EBRT and myeloablative therapy [336,337] alterations XRCC3 and MSH2 [415]
Wilms tumor	alterations of WT1, CTNNB1, WTX, TP53, and MYNC [416] syndromes including WAGR, Drash, Beckwith- Wiedemann, Sotos, Perlman, Edward’s, Frasier, BLM, LFS, and Simpson-Golabi- Behmel	combination of surgery and CT comprising vincristine, dactinomycin, and doxorubicin in children with localized tumors plus EBRT for high-risk patients in metastatic disease [417,418] TRK inhibitors and anti-PD-1-antibodies (nivolumab, pembrolizumab) for advanced treatment-refractory renal cell carcinoma might be implemented into therapeutic strategies for Wilms tumors [419,420]	cardiotoxicity, musculoskeletal, growth and development, radiogenic lung fibrosis [417,418,421]	bone and soft-tissue sarcomas, breast cancer, lymphoma, leukemia, melanoma [417]	anthracyclines (doxorubicin) and EBRT [417,418,421]
Retinoblastoma	mutation of RB1 [422]	enucleation, intravenous CT (melphalan with or without topotecan and/or carboplatin), focal therapy (laser therapy, cryotherapy), intra-arterial CT with focal therapy [423]	loss of vision in the affected eye, deformities in the bones around the eye, myocardial dysfunction, hypothyroidism [424,425]	leiomyosarcoma, osteosarcoma, melanoma, cancer of the lung, bladder, brain, and nasal cavities [424,425,426]	germline RB1 mutation and EBRT [424,425,426]

*Abbreviations*: SPM, second primary malignancy; BLM, Bloom syndrome; LFS, Li-Fraumeni syndrome; CT, chemotherapy; EBRT, external beam radiotherapy.

## Data Availability

No novel data were generated in this study. Data sharing does not apply to this article.

## Abbreviations

3D-CRT	3-Dimensional conformal radiotherapy
ABVD	Adriamycin [doxorubicin], bleomycin, vinblastine, and dacarbazine
ACT	Adaptive T-cell therapy
ACVR1	Activin A receptor type I
AIEOP	Assoziazione Italiana Ematologica Oncologia Pediatrica
AKT	Protein kinase B
ALK	Anaplastic lymphoma kinase
ALL	Acute lymphoblastic leukemia
AML	Acute myeloid leukemia
AML1	Acute myeloid leukemia 1
ASPL	Alveolar soft part sarcoma locus
AT	Ataxia-telangiectasia
ATRX	Chromatin remodeler ATP-dependent helicase
BCL2	B-cell lymphoma 2
BCOR	BCL-6 corepressor
BET	Bromodomain and extra-terminal motif
BFM	Berlin-Frankfurt-Muenster
BLM	Bloom syndrome
BRAF	v-raf murine sarcoma viral oncogene homolog B
BRCA2	Breast cancer 2
BTK	Bruton tyrosine kinase
CAR	Chimeric antigen receptor
CASP8	Caspase 8
CBFB	Core-binding factor subunit beta
CD	Cluster of differentiation
CDC5L	Cell division cycle 5-like
CDK	Cyclin-dependent kinase
CDKN2	Cyclin-dependent kinase inhibitor
CHK1	Checkpoint kinase 1
CHK2	Checkpoint kinase 2
CLL	Chronic lymphocytic leukemia
CNS	Central nervous system
CT	Chemotherapy
CML	Chronic myelogenous leukemia
CTLA-4	Cytotoxic T lymphocyte antigen-4
CTNNB1	Catenin beta-1
CXCL12	C-X-C motif chemokine 12
CXCR4	C-X-C chemokine receptor type 4
CYP	Cytochrome P450
DNA	Deoxyribonucleic acid
DNA-PK	DNA-dependent protein kinase
DIPG	Diffuse intrinsic pontine glioma
DS	Down syndrome
EBV	Epstein-Barr-Virus
EBRT	External beam radiation therapy
EGFR	Epidermal growth factor receptor
ERCC	Excision repair cross-complementing
ERG	ETS-related gene
ERK	Extracellular signal-regulated kinase
EPN	Ependymoma
ES	Ewing sarcoma
EZH2	Enhancer of zeste homolog 2
FA	Fanconi anemia
FBXW7	F-box/WD repeat-containing protein 7
FGFR	Fibroblast growth factor receptor
FLI-1	Leukemia integration 1 transcription factor
FLT3	fms like tyrosine kinase 3
FOXO1	Forkhead box protein O1
GCCR	German Childhood Cancer Registry
GST	Glutathione s-transferase
HDAC	Histone deacetylase
HER2	Human epidermal growth factor receptor 2
HGG	High-grade glioma
HIC1	Hypermethylated in cancer 1
HL	Hodgkin lymphoma
HSCT	Hematopoietic stem cell transplantation
ICI	Immune checkpoint inhibitor
IDH	Isocitrate dehydrogenase isozymes
IFN	Interferon
IGF1R	Insulin-like growth factor 1 receptor
IGRT	Image-guided radiotherapy
IKZF1	Ikaros family zinc finger protein 1
IL-2	Interleukin 2
IMRT	Intensity-modulated radiotherapy
IR	Ionizing radiation
ITD	Internal tandem duplication
IVA	Ifosfamide, vincristine, and actinomycin D
JAK2	Janus kinase 2
KRAS	Kirsten rat sarcoma viral oncogene
KIT	Tyrosine-protein kinase KIT
LFS	Li–Fraumeni syndrome
LGG	Low-grade gliomas
LIG4	DNA ligase 4
LSD1	Lysine-specific demethylase 1
MAPK	Mitogen-activated protein kinase
MB	Medulloblastoma
MDS	Myelodysplastic syndrome
MET	Mesenchymal-epithelial transition factor
MGMT	O^6^-methylguanine DNA methyltransferase
MLL	Mixed-lineage leukemia
MOPP	Mustargen [mechlorethamine], oncovin [vincristine], procarbazine, and prednisone
MSH	MutS homolog
MYC	bHLH transcription factor
mTOR	Mechanistic target of rapamycin
NB	Neuroblastoma
NBS	Nijmegen breakage syndrome
NF	Neurofibroma
NF1	Neurofibromatosis 1
NF2	Neurofibromatosis 2
NF-kB	Nuclear factor ‘kappa-light-chain-enhancer’ of activated B-cells
NHL	Non-Hodgkin lymphoma
NKX2.2	Homeobox protein Nkx-2.2
NQO1	Nicotinamide adenine dinucleotide phosphate: quinone oxidoreductase
NRMS	Nonrhabdomyosarcoma
NTRK	Neurotrophic receptor tyrosine kinase
OP/EPA	Vincristine, prednisone, procarbazine/etoposide, and doxorubicin
OPPA/COPP	Cyclophosphamide, vincristine, prednisone, and procarbazine
OS	Osteosarcoma
OTX2	Orthodenticle homeobox 2
PALB2	Partner and localizer of BRCA2
PAPPA	Pregnancy-associated plasma protein A
PARP	Poly(ADP-ribose)-polymerase
PAX3	Paired box gene 3
PD-1	Programmed cell death protein 1
PDGFR	Platelet-derived growth factor receptor
PD-L1	Programmed cell death protein ligand 1
PI3K	Phosphatidylinositol 3-kinase
PIK3CA	Phosphatidylinositol-4,5-bisphosphate 3
PIM1	Proto-oncogene serine/threonine-protein kinase Pim-1
PMP22	Peripheral myelin protein 22
PRIM1	DNA primase small subunit
PTCH1	Patched homolog 1
PTEN	Phosphatase and tensin homolog
PVT1	Plasmacytoma variant translocation 1
RAG1	Recombination activating gene 1
RARA	Retinoic acid receptor alpha
RAS	Rat Sarcoma viral oncogene
RASSF1A	Ras association domain-containing
RB	Retinoblastoma
RMS	Rhabdomyosarcoma
RNA	Ribonucleic acid
RR	Relative risk
R/R	Relapsed/refractory
RUNX1	Runt-related transcription factor 1
SBRT	Stereotactic body radiotherapy
SEER	Surveillance, epidemiology and end results
SHH	Sonic hedgehog
SIR	Standardized incidence ratio
SKP2	S-phase kinase-associated protein 2
SMARC	SWI/SNF-related matrix-associated actin-dependent regulator of chromatin subfamily
SMR	Standardized mortality ratio
SPM	Second primary malignancy
STS	Soft tissue sarcoma
SUFU	Suppressor of fused homolog
TFE3	Transcription factor E3
TMZ	Temozolomide
TNF	Tumor necrosis factor
TRK	Tyrosine receptor kinase
TP53	Tumor suppressor protein 53
TPMT	Thiopurine methyltransferase
TPCV	Thioguanine, procarbazine, lomustine, and vincristine
TSC	Tuberous sclerosis complex
VAC	Vincristine, actinomycin D, and cyclophosphamide
VACD	Vincristine, cyclophosphamide, actinomycin D, and doxorubicin
VEGF	Vascular endothelial growth factor
WRN	Werner syndrome
XPD	Xeroderma pigmentosum group D gene
XRCC1	X-ray cross-complementing factor 1
YAP1	Yes-associated protein 1
